# Identification of FDA-approved IMPDH inhibitors boosting the broad-spectrum antiviral potential of ribavirin through depleting cellular GTP pool

**DOI:** 10.1016/j.apsb.2026.05.017

**Published:** 2026-05-20

**Authors:** Qiangyun Ai, Huina Hu, Kaixiong Ma, Qingxing Wang, Shenglin Huang, Yongzhi Lu, Nan Qi, Jinsai Shang, Ruikun Du, Shaobo Wang

**Affiliations:** a State Key Laboratory of Respiratory Disease, The First Affiliated Hospital of Guangzhou Medical University, Guangzhou National Laboratory Clinical Base, School of Basic Medical Sciences, Guangzhou Medical University, Guangzhou 511436, China; b Guangzhou National Laboratory, Department of Basic Research, Guangzhou 510005, China; c Wuhan Institute of Virology, Chinese Academy of Sciences, Wuhan 430071, China; d Qingdao Academy of Chinese Medical Sciences, Shandong University of Traditional Chinese Medicine, Qingdao 266122, China; e University of the Chinese Academy of Sciences, Beijing 100049, China

**Keywords:** Synergistic screening, FDA-approved drugs, Ribavirin, Mycophenolate mofetil, IMPDH, GTP pool, Misincorporation, Broad-spectrum synergy

## Abstract

Nucleoside analogues are among the most promising broad-spectrum antiviral agents. We developed a high-throughput synergistic screening system to identify US Food and Drug Administration-approved drugs that enhance the effectiveness of nucleoside analogues. Unlike conventional combination approaches, this strategy does not require boost agents to have direct antiviral activity; instead, they can enhance antiviral agent’s effect through novel synergistic mechanisms. We identified mycophenolate mofetil (MMF), which significantly boosts ribavirin’s (RBV) antiviral efficacy against influenza A virus, even at concentrations where MMF alone shows minimal activity. MMF enhances RBV by inhibiting IMPDH (inosine-5′-monophosphate dehydrogenase) and reducing cellular GTP levels, potentially increasing RBV incorporation and its antiviral potency. This mechanism was confirmed by adding exogenous GTP, which reversed the synergy. In a mouse model, MMF also enhanced RBV’s effectiveness against PR8 *in vivo*. Moreover, MMF showed synergistic effects against other influenza subtypes and RNA viruses including ZIKV and HCoV-OC43, demonstrating the potential wide applicability of this strategy. Our study introduces a new screening system and identifies novel RBV combinations, offering a promising approach for developing effective broad-spectrum antiviral combinations against emerging viral threats.

## Graphical abstract

This study demonstrates that the MMF suppresses intracellular GTP production, thereby promoting the incorporation of RBV-TP into the viral genome and enhancing the broad-spectrum antiviral activity of ribavirin.

**Figure:**
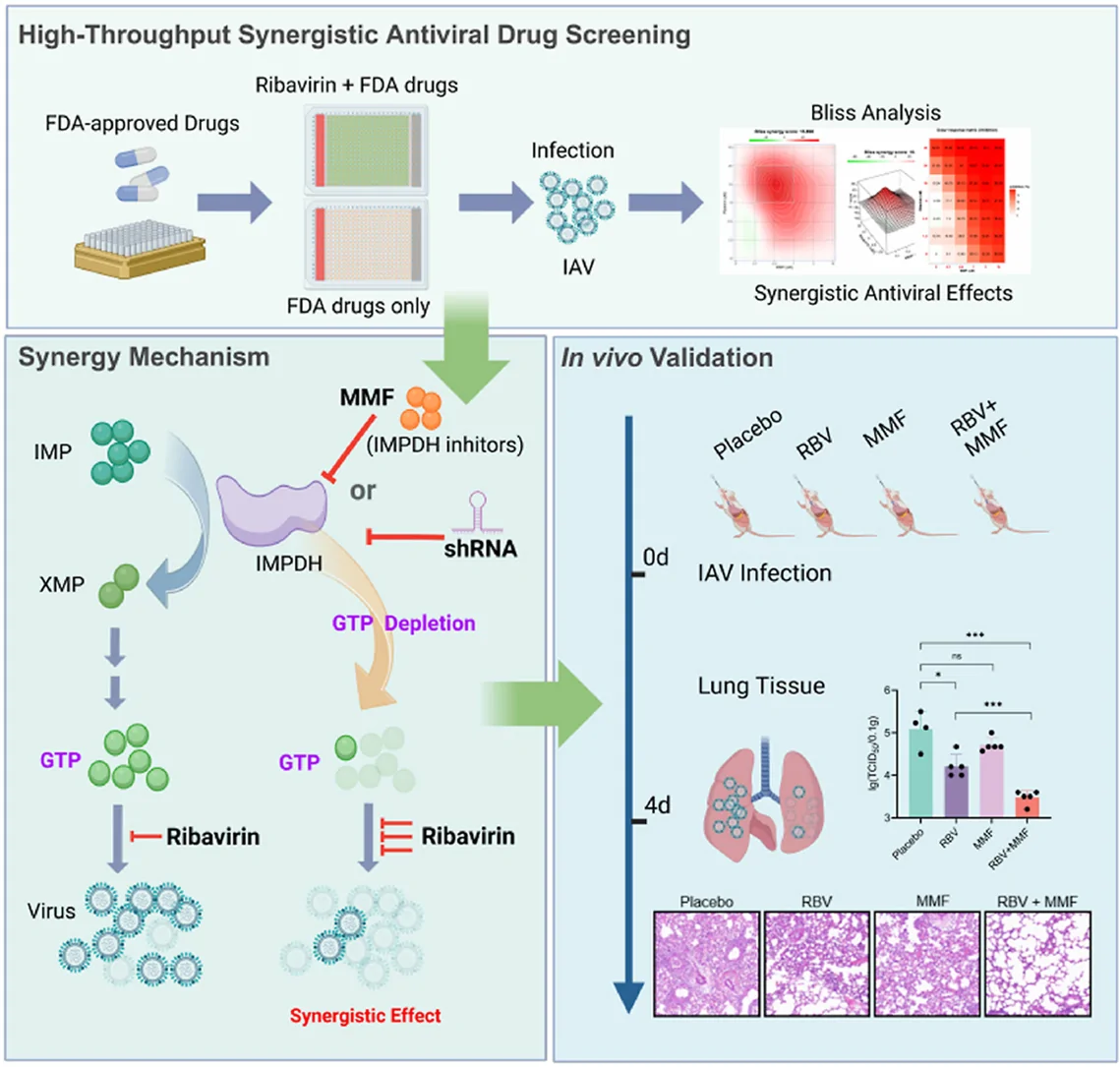

## 1 Introduction

Emerging and re-emerging viral infectious diseases continue to pose a significant threat to human society. The etiological agents include SARS-CoV-1 (severe acute respiratory syndrome coronavirus 1)^1^, ^2^, ^3^, MERS-CoV (Middle East respiratory syndrome coronavirus)^4^, SARS-CoV-2 (severe acute respiratory syndrome coronavirus 2)^5^^,^^6^, ZIKV (Zika virus)^7^^,^^8^, influenza A virus (H1N1^9^, H5N1^10^, ^11^, ^12^, H3N2^13^ subtypes). In the case of SARS-CoV-2, it has caused a global pandemic resulting in over 6.9 million fatalities and profound societal consequences^14^. In addition to coronaviruses, influenza viruses continuously circulate and threaten human health. Since 2024, the highly pathogenic avian influenza H5N1 has spilled over from animals to humans frequently, leading to more than 25 confirmed human infection cases. The incidence of such outbreaks has markedly increased over the last two decades, with the causative viruses originating from diverse families. This highlights an urgent need for the development of broad-spectrum antiviral interventions.

Nucleoside analogues are among the most promising agents and have the potential to inhibit multiple viruses. For instance, ribavirin (RBV), a synthetic nucleoside analogue, functions as a competitive inhibitor of guanosine triphosphate (GTP)^15^, ^16^, ^17^. It incorporates into the viral RNA genome, thereby disrupting replication fidelity. Clinically, RBV has been approved for the treatment of chronic hepatitis C virus (HCV)^18^, ^19^, ^20^, severe respiratory syncytial virus infections^21^^,^^22^, and certain hemorrhagic fevers. However, as a broad-spectrum agent, its inhibitory efficacy varies significantly across different viral families. We aim to identify a compound that synergizes with RBV to enhance its antiviral potential by targeting cellular pathways. This strategy differs from conventional synergistic antiviral combinations, which typically consist of two inhibitors, both possessing direct antiviral activity. Such combinations are designed to enhance antiviral efficacy and prevent drug resistance. Our approach goes beyond this conventional strategy, as the compound does not necessarily need to exhibit direct antiviral activity but instead enhances RBV’s antiviral effect through a novel synergistic mechanism. This approach will substantially broaden the scope of potential synergistic compounds.

Interestingly, a recent study demonstrated that dihydroorotate dehydrogenase (DHODH) inhibitors alone do not inhibit SARS-CoV-2 but significantly enhance the antiviral efficacy of a nucleoside analogue molnupiravir *in vivo*^23^. Another well-known example is Paxlovid, which consists of the combination of nirmatrelvir and ritonavir. While ritonavir lacks direct antiviral activity, it enhances the efficacy of nirmatrelvir by slowing its metabolism and prolonging its half-life in human^24^. However, these synergistic effects were not identified through systematic screening approaches. Consequently, synergistic inhibitors with no direct antiviral potential are often overlooked in drug discovery processes.

Inspired by these studies, we aimed to develop a high-throughput screening approach to identify compounds that exhibit synergy with nucleoside analogues, even lack direct antiviral activity. In this work, we established a synergistic antiviral drug screening system to identify synergistic drugs with RBV from an FDA-approved drug library. Through systematic screening and validation, we identified multiple candidates with synergistic potential. Mycophenolate mofetil (MMF) was selected for further investigation. The results showed that MMF synergizes with RBV by acting on its cellular target IMPDH (inosine-5′-monophosphate dehydrogenase) and depleting cellular GTP pool, therefore potentially increasing RBV incorporation efficiency and leading to stronger inhibitory potency. We further confirmed that MMF alone exhibits limited antiviral activity *in vivo*; however, it significantly enhances the antiviral efficacy of RBV against influenza virus. Furthermore, MMF was demonstrated to synergize with RBV against other influenza subtypes as well as other RNA viruses, such as human coronavirus OC43 (HCoV-OC43) and ZIKV, illustrating the potential broad applicability of this synergistic strategy with RBV. Our work provides a novel synergistic screening system and identifies new candidates that synergize with RBV, representing an unexplored strategy to develop innovative broad-spectrum antiviral interventions.

## 2 Materials and methods

### 2.1 Cells

A549 (human lung adenocarcinoma epithelial cells), HEK293T (derived from human embryonic kidney), and MDCK (Madin-Darby canine kidney cells) were commercially obtained from the American Type Culture Collection (ATCC) and Huh 7 (hepatoma cells) was maintained in our laboratory. These cell lines were cultured in DMEM (Gibco) supplemented with 10% (*v*/*v*) FBS (Sigma), 100 U/mL penicillin, and 100 μg/mL streptomycin.

### 2.2 Viruses

The recombinant PR8-NS1-FLuc and PR8-NS1-GFP viruses (A/Puerto Rico/8/1934, H1N1), which expresses firefly luciferase and green fluorescent protein as reporter genes respectively, were constructed and propagated as previously described^25^. The H3N8-GFP strain (A/China/ZMD-22-2/2022, H3N8) engineered to express GFP was kindly provided by Dr. Nan Qi. These strains were propagated by inoculating 9- to 12-day-old specific pathogen-free (SPF) chicken embryos. The CA07-GFP (A/California/08/2009, H1N1) strain is stored in our laboratory, while the H3N2-GFP strain (A/China/LZP/2017, H3N2) engineered to express GFP was also provided by Dr. Nan Qi. These strains were propagated in MDCK cells with 3 μg/mL TPCK-trypsin. ZIKV-ZsGreen reporter virus was rescued as described previously^26^, the infectious clone was a gift from Dr. Andres Merits at University of Tartu. HCoV-OC43 was purchased from ATCC (VR-1558). Both ZIKV-ZsGreen and HCoV-OC43 were propagated in Vero cells. All virus stocks were stored at −70 °C until use.

### 2.3 Compounds and chemical libraries

The RNA-dependent RNA polymerase inhibitor RBV (ribavirin, T0684, purity: 99.83%), MMF (mycophenolate mofetil, T6293, purity: 99.85%), and IMPDH inhibitors merimepodib (VX-497, T3444, purity: 99.31%), AVN-944 (VX-944, T1979, purity: 99.3%), mizoribine (T1188, purity:99.95%), and OSP (oseltamivir phosphate, T1486, purity: 99.94%) were obtained from TargetMol. The FDA Drug Library (Approved & Pharmacopeial, L1300) was purchased from Selleck Chemicals. All compounds used in this study were of ≥99% purity, as confirmed by the manufacturer’s certificate of analysis. Compounds were initially diluted to a concentration of 50 μmol/L in medium containing 1% DMSO.

### 2.4 High-throughput synergistic screens with RBV against IAV

Synergy screening necessitates both dual-drug and single-drug screenings to generate a ranked list of synergy scores through systematic analysis and calculation. The detailed procedures are outlined below. 5000 cells of A549 per well were seeded into a W384-well TC-treated black opaque cell plate with a volume of 20 μL per well. After overnight incubation to allow cell adherence, the supernatant was discarded. Each well received 20 μL of DMEM containing 2% FBS and either 10 μmol/L RBV or 1% DMSO. Next, 10 μL of FDA-approved drugs were added to columns 3 to 22 of the W384-well plate. Columns 1 and 2 served as negative controls and received 10 μL of DMEM containing 1% DMSO and 2% FBS. Columns 23 and 24 as positive controls and were treated with 10 μL of CPDA. Following a 6-h incubation period, 20 μL of PR8-NS1-FLuc virus stock (MOI = 0.5) was added to each well. At 24 h post-infection, 20 μL of one-step luciferase substrate was added to each well, and chemiluminescence values were measured using a PE microplate reader. The *Z*′-factor values were calculated based on the negative and positive controls.

According to the Bliss independence model^27^, the synergy score was calculated as follows. First, determining the additive effect of the two drugs in the absence of synergy by calculating *E*_A_+*E*_B_‒*E*_A_*E*_B_, where *E*_A_ represents the inhibition rate of RBV alone and E_B_ represents the inhibition rate of the FDA-approved drug alone. Next, comparing the observed inhibition rate from the dual-drug cell experiment (*E*_A,B_) with the theoretically additive inhibition rate *E*_A_+*E*_B_‒*E*_A_*E*_B_. If the difference between the observed inhibition rate and the additive inhibition rate *S*_Bliss_ is greater than zero as Eq. (1):

(1) *S*_Bliss_ = *E*_A,B_ ‒ (*E*_A_+*E*_B_*‒E*_*A*_*E*_B_)

It indicates a synergistic effect between the two drugs, suggesting that the FDA-approved drug can enhance the antiviral activity of RBV against the influenza virus. Conversely, if this difference is less than zero, it suggests an antagonistic effect, while a difference equal to zero indicates an independent or additive effect.

There is no universally accepted threshold to define a standard synergy score because combinatorial synergy is highly context dependent. However, empirical evidence suggests that a synergy score close to 0 provides limited confidence in either synergy or antagonism. Therefore, we can generally interpret the synergy scores as follows: when the synergy score is less than −10, the interaction between the two drugs is likely to be antagonistic; between −10 and 10, the interaction is likely to be additive; greater than 10, the interaction is likely to be synergistic. Here, we set a synergy score of 10 as our baseline and further refine this threshold based on the number of drug targets screened.

### 2.5 Candidates ranking and re-confirmation screen

Based on the calculated synergy scores, we sorted the drug candidates and generated a bubble chart to identify those exceeding the threshold. We then treated the cells with these selected candidates, measured the luminescence values following PR8-NS1-FLuc reporter virus infection, and calculated the inhibition rates. Using Bliss independence analysis, we verified their synergistic antiviral effects in combination with RBV. A synergy matrix is utilized to perform a synergistic antiviral inhibition analysis on these selected drugs. The predefined threshold is applied to score the most synergistic area (MSA) as determined by Bliss analysis, which corresponds to the 5 × 5 dose window with the highest synergy within the dose-response matrix. This approach facilitates the identification of the concentration range exhibiting the strongest synergistic effects.

### 2.6 Two-dimensional representation of dose response interaction matrix

A systematic concentration matrix of RBV and MMF was designed to evaluate combinatorial effects. RBV and MMF solutions were prepared in five concentrations using a threefold dilution series, with maximum concentrations of 90 and 30 μmol/L, respectively. A zero-concentration gradient was included for each drug to serve concurrently as a single-drug control when paired with active concentrations of the counterpart drug and as a virus-infected positive control when both drugs were absent. A549 or Huh 7 cells were seeded in 96-well plates at 20,000 cells/well and incubated overnight. The drug concentration matrix was applied to cells for 6 h before infection with PR8-NS1-FLuc or PR8-NS1-GFP virus at an MOI of 0.5. Antiviral activity was assessed at 24 h post-infection by measuring either luciferase activity (for PR8-NS1-FLuc) or *via* high-content imaging (for PR8-NS1-GFP). The antiviral inhibition rate (%) for each drug combination was calculated and antiviral rates were normalized to cell number based on DAPI-stained nuclei counts. Theoretical additive inhibition rates were derived from single-agent dose-response curves. Synergy scores were calculated using the Bliss independence model as Eq. (1). The resulting synergy scores were compiled into a matrix for visualization and analysis^28^^,^^29^.

### 2.7 Cell viability evaluated by CCK8

In accordance with the synergy screening protocol, cell viability was assessed concurrently following drug treatment. The CCK8 kit was utilized. According to the protocol, 48 h post-drug addition, the CCK8 reagent was introduced into the cell supernatant and incubated at 37 °C for 1 h. Subsequently, the OD value were measured using a microplate reader. The percentage of cell viability was generated, and a dose-response curve was made to assess the cytotoxicity.

### 2.8 Exogenous GTP supplementation assay

A549 cells were seeded into 96-well plates at a density of 20,000 cells per well and incubated overnight. The cells were then treated with single or combined concentrations of RBV and MMF. In parallel, 100 μmol/L rNTPs were added to designated wells to assess their potential modulatory effects. Following a 6-h incubation period, the cells were infected with either PR8-NS1-FLuc or PR8-NS1-GFP virus at a multiplicity of infection (MOI) of 0.5. At 24 h post-infection, antiviral activity was quantified by measuring chemiluminescence (for PR8-NS1-FLuc) or GFP expression (for PR8-NS1-GFP) to determine whether exogenous GTP supplementation could rescue the antiviral synergy between RBV and MMF.

### 2.9 IMPDH2 knockdown and synergy of antiviral with RBV

Three specific shRNAs targeting the host *Impdh2* gene were designed and cloned into the pLKO.1 lentiviral vector (Supporting Information Table S2). These constructs were subsequently packaged into lentiviral particles, which were then used to transduce A549 and Huh 7 cells. After 48 h of puromycin selection, the surviving cells were collected for Western blot analysis or quantitative real-time PCR to evaluate IMPDH2 protein expression or mRNA levels with primers listed in Supporting Information Table S1, thereby verifying the knockdown efficiency of IMPDH2.

A549 cells were divided into four experimental groups: (1) wild-type cells treated with RBV, (2) IMPDH2-knockdown cells, (3) untreated wild-type cells (control), and (4) IMPDH2-knockdown cells treated with DMSO (vehicle control). All cell groups were infected with either PR8-NS1-FLuc or PR8-NS1-GFP influenza virus at a multiplicity of infection (MOI) of 0.5. Viral replication was assessed at 24 h post-infection by measuring chemiluminescence (for PR8-NS1-FLuc-infected cells) or quantifying GFP fluorescence intensity (for PR8-NS1-GFP-infected cells) using a microplate reader or fluorescence microscopy, respectively. This experimental setup allowed evaluation of potential synergistic antiviral effects between IMPDH2 knockdown and RBV treatment.

To investigate whether exogenous GTP supplementation could reverse the observed synergistic inhibition, the same infection and quantification protocol was repeated with IMPDH2-knockdown cells supplemented with varying concentrations of GTP.

### 2.10 Next-generation sequencing for viral genome mutagenesis analysis

To enable attachment, Huh 7-TMPRSS2 cells were plated in 6-well plates and incubated overnight. The adhered cells were then exposed to DMSO, RBV, MMF, or a combination of RBV and MMF. Six hours post-treatment, infection was carried out using the PR8-NS1-GFP strain at a MOI of 0.5. After a 24-h infection period, both cells and culture supernatant were harvested as the first virus passage (P1). Absolute quantification of the PR8 N gene was performed on 2 μL of P1 supernatant *via* one-step qRT-PCR employing the HiScript II SYBR Green kit.

Under sustained drug pressure, virus was serially passaged for three rounds (P1 through P3) using normalized inocula. Following the third passage, P3 materials were collected. Total RNA was isolated from the harvested P3 cells using Trizol reagent for subsequent next-generation sequencing. Ribosomal RNA was removed from the total RNA, after which cDNA was generated with random hexamers and reverse transcriptase. The resulting cDNA was processed through end-repair, adenine-tailing, and adapter ligation. Post size selection, the fragments were PCR-amplified. The amplification products were purified with AMPure XP beads to construct strand-specific libraries suitable for paired-end sequencing.

Initial quality control of the raw sequencing reads was conducted with FastQC, followed by adapter and quality trimming using Trim Galore. High-quality reads were then mapped to the reference viral genome with BWA. Variant identification and analysis were executed using SAMtools and BCFtools. All subsequent statistical analyses and data visualization were implemented in the R programming environment.

Concurrently, the TCID_50_ assay was utilized to determine viral titers in the P2 supernatants. Specific infectivity, expressed as the ratio of TCID_50_ titer to genome copy number, was calculated for each drug treatment condition based on these measurements.

### 2.11 Mouse experiments

Female BALB/c mice aged 6 to 8 weeks were utilized in this study. The experimental design included a control group (Placebo: PBS), single treatment groups (RBV at 40 mg/kg/dose, administered twice daily; MMF at 15 mg/kg/dose, administered twice daily), and a combined treatment group (RBV+MMF, with the same dosing volume as the single drug groups). All treatments were administered *via* oral gavage at a volume of 200 μL. Subsequently, mice were inoculated intranasally with 50 μL of virus dilution containing 1000 PFU of PR8 virus. Weight changes were monitored and recorded throughout the experiment. For the *in vivo* survival experiment, an extended observation period lasting up to 14 days post-infection was performed, both body weight changes and mortality of mice were closely monitored. In addition, a group treated with oseltamivir phosphate at 10 mg/kg/dose (b.i.d.) was included as a standard-of-care positive control. The viral load in lung tissue was quantified using the TCID_50_ method and viral genome copies were quantified by qPCR with the primers listed in Table S1. Histopathological changes and the localization of viral antigens in lung tissue were assessed through pathological tissue sections and immunohistochemical staining of the influenza virus NP protein. Additionally, the expression levels of cytokines associated with antiviral innate immunity and inflammation, including *Ccl2*, *Isg 15*, *Il-1*, *Cxcl10*, *Mx1*, and *Tnfα*, were analyzed by qPCR with the primers listed in Table S1.

### 2.12 Statistical analyses

The data are presented as mean ± standard deviation of the mean (SD). For two-group comparisons, an unpaired two-tailed Student’s *t*-test was employed. For comparisons among three or more groups, one-way ANOVA was performed (ns *P* > 0.05, ∗*P* < 0.05, ∗∗*P* < 0.01, ∗∗∗*P* < 0.001, ∗∗∗∗*P* < 0.0001). All statistical analyses were conducted using GraphPad Prism 9 software (version 9.5) or R software (version 4.2.1), with visualizations created using GraphPad Prism 9 software or R ggplot 2 packages.

### 2.13 Data availability

All raw and processed data generated from this study will be made publicly available, adhering to appropriate data sharing policies and guidelines, to facilitate reproducibility and further research in the field.

### 2.14 Ethics

All experiments involving virus were conducted in the biosafety level 2 (BSL-2) and animal biosafety level 2 (ABSL-2) facilities in Guangzhou National Laboratory (GZNL). All animal experiments were approved by the Ethics Committee of Guangzhou National Laboratory (GZLAB-AUCP-2024-03-A08).

## 3 Results

### 3.1 High-throughput screening identified FDA-approved drugs boosting the antiviral potential of ribavirin against influenza A virus

We aimed to identify inhibitors that exhibit synergistic effects with RBV, a nucleoside analogue, regardless of whether they possess direct antiviral activity. Antiviral synergy is defined as the interaction between two compounds producing an effect greater than their additive combination. To achieve this, we developed a high-throughput screening system to evaluate the synergistic potential of RBV in combination with library compounds, while also screening the library compounds alone in parallel (Fig. 1A). After quantifying the inhibition percentages, we applied Bliss analysis to assess whether the two drugs interact. There are three possibilities: (a) A synergy score of zero indicates an additive effect; (b) A score greater than zero suggests a positive interaction, resulting in synergy; (c) A score less than zero implies an antagonistic effect.

**Figure 1:**
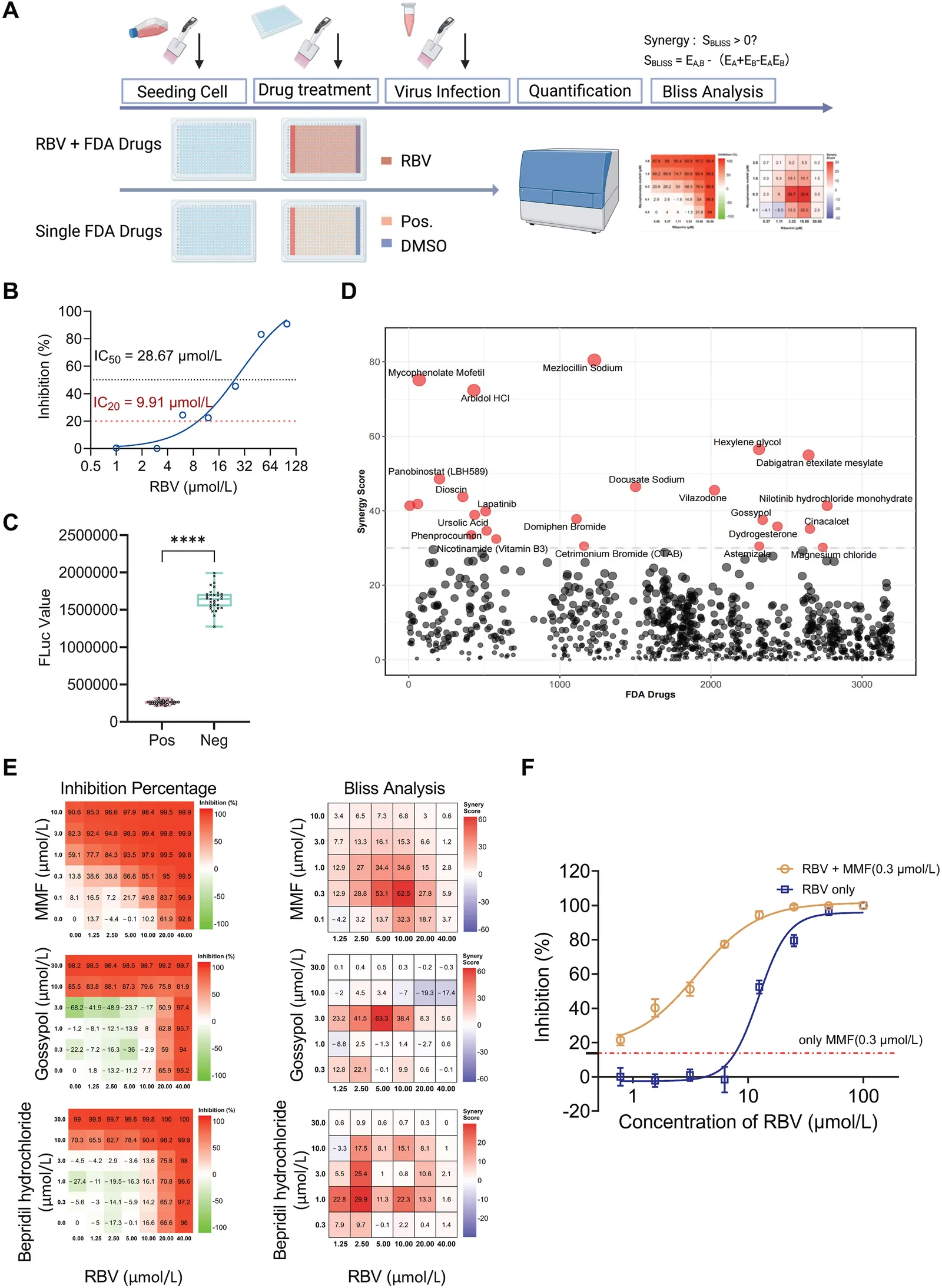
A high-throughput synergistic screening identified FDA-approved drugs boosting the antiviral potential of RBV. (A) Strategy diagram for screening synergistic combinations. To meet the requirements of Bliss analysis, the antiviral efficacy of individual FDA-approved drugs and dual-drug combinations (RBV + FDA) was evaluated in parallel. (B) The IC_20_ of RBV against PR8-NS1-FLuc. A549 cells were treated with varying concentrations of RBV for 6 h, subsequently infected with PR8-NS1-FLuc at an MOI of 0.5, and luminescence was measured at 36 h post-infection. The inhibition percentages against PR8-NS1-FLuc were calculated, and the corresponding IC_20_ value was determined and indicated. (C) Luciferase (Luc) values from positive and negative controls were measured during the synergistic screen, and these values were used to calculate the corresponding *Z′*-factor. *n* = 32 independent biological replicates. ∗∗∗∗*P* < 0.0001, unpaired two-tailed Student’s *t*-test. (D) The results of high-throughput synergistic screening were visualized in a bubble chart, with the *y*-axis representing the synergy score. (E) Synergy matrix and analysis for the three synergistic drug combinations. Antiviral inhibition percentages at various concentrations of the two drugs were measured. A549 cells were treated with different concentration combinations of RBV and MMF/Gossypol/Bepridil, followed by infection with PR8-NS1-FLuc for 36 h. A synergy matrix was utilized to evaluate the synergistic antiviral inhibitory effects. (F) Inhibition curves for A549 cells treated with RBV and the selected candidate. A549 were treated with DMSO, RBV alone, MMF alone (0.3 μmol/L) or RBV with MMF (0.3 μmol/L), followed by infection with PR8-NS1-FLuc for 36 h. The inhibition percentages were determined by measuring the luciferase activity.

Firstly, we utilized a reporter influenza A virus H1N1 strain (PR8-NS1-FLuc) carrying a reporter gene, firefly luciferase, to evaluate the antiviral activity of RBV based on measuring luciferase activity. This approach enabled us to determine the IC_50_ and IC_20_ values of RBV (Fig. 1B) within our screening system. To maximize the identification of potential synergistic candidates, we selected RBV at its IC_20_ concentration of 10 μmol/L (Supporting Information Fig. S1A) for the screen, as synergy evaluation becomes constrained when the inhibition rate of ribavirin alone exceeds 50%. We performed this high-throughput screen using an FDA-approved drug library comprising 3200 compounds at a concentration of 10 μmol/L in a 384-well plate format (Fig. S1B). Each 384-well plate included 32 control wells (16 positive and 16 negative controls) to ensure the robustness and reliability of the screening process, which was further validated by calculating the *Z*′-factor (Fig. 1C). If the *Z*′ factor is above 0.5 indicating a robust screening. Following the screening of the 3200 FDA-approved compounds, we determined both the inhibition rates of single-library compounds and the combination of RBV with library compounds. The *Z*′-factor values across the 10 plates ranged from 0.58 to 0.75 (Fig. S1C), confirming the reliability of the screening system. Therefore, we further assessed the synergistic effects of drug combinations using Bliss analysis (Fig. S1D). The synergy score based on the Bliss independence model was calculated as follows. First, the theoretical inhibition rate for an additive effect is determined using the formula *E*_A_ *+ E*_B_*‒E*_A_*E*_B_, where *E*_A_ and *E*_B_ represent the inhibition rates of RBV alone and the FDA-approved drug alone, respectively. The synergy score is then obtained by subtracting this theoretical additive value from the experimentally observed inhibition rate of the drug combination (*E*_A,B_). Thus, the Bliss synergy score is defined as Eq. (1), with positive, zero, negative values indicating synergistic, additive, and antagonistic effects, respectively. Candidates were ranked according to their synergy scores, and the top-ranked candidates were selected for further validation (Fig. 1D). A549 cells were treated with multiple concentrations of each drug, both individually and in combination, generating a matrix of 30 drug pairs. The infection levels were quantified by the luciferase activity. Subsequently, we confirmed that three compounds—mycophenolate mofetil (MMF), gossypol, and bepridil hydrochloride—exhibited significant synergy when combined with RBV (Fig. 1E). Especially for MMF, the inhibition rate for 0.3 μmol/L MMF monotherapy was 13.8%, but we observed a notable reduction in viral infection upon co-treatment at multiple concentrations (Fig. 1F). Furthermore, isobologram analysis revealed that RBV and MMF act synergistically against PR8-NS1-FLuc in A549 cells (Fig. S1E). Taken together, we developed a high-throughput screening approach to identify FDA-approved compounds that exhibit synergy with RBV, regardless of whether they possess direct antiviral activity. Multiple compounds were confirmed to synergize with RBV, among which MMF demonstrated the highest potential.

### 3.2 Mycophenolate mofetil synergized with ribavirin through depleting the intracellular GTP pool

MMF is an inhibitor of inosine monophosphate dehydrogenase (IMPDH) and disrupts *de novo* purine synthesis (Fig. 2A). In the preliminary validation, MMF exhibited significant synergistic activity with RBV. Individually, RBV (10 μmol/L) and MMF (0.3 μmol/L) showed negligible antiviral effects in A549 cells. However, their combination markedly improved antiviral efficacy, achieving an inhibition percentage of approximately 80% and a synergy score of 30.4. Furthermore, at higher concentrations (20 μmol/L RBV and 0.3 μmol/L MMF), viral replication was almost completely suppressed (Fig. 2B). We further confirmed this synergy in another susceptible cell line, Huh 7 (human hepatocyte), to rule out cell-dependency (Fig. 2C). Isobologram analysis revealed that RBV and MMF act synergistically against PR8-NS1-GFP in Huh7 cells (Fig. S1F). Given that the host target of MMF is IMPDH, we further investigated whether other IMPDH inhibitors with distinct chemical scaffolds, such as AVN-944^30^, VX497^31^, and mizoribine^32^ (Fig. 2D), exhibit comparable synergistic activity with RBV. To this end, we conducted a 5 × 5 dose-response analysis in A549 cells infected with the PR8 reporter virus. Our results revealed significant synergy between those IMPDH inhibitors (AVN-944, VX497 and mizoribine) and RBV (Fig. 2E, Supporting Information Fig. S2A‒C). Notably, this synergy was observed at submicromolar concentrations for AVN-944 and VX497, while mizoribine exhibited synergy within the micromolar range.

**Figure 2:**
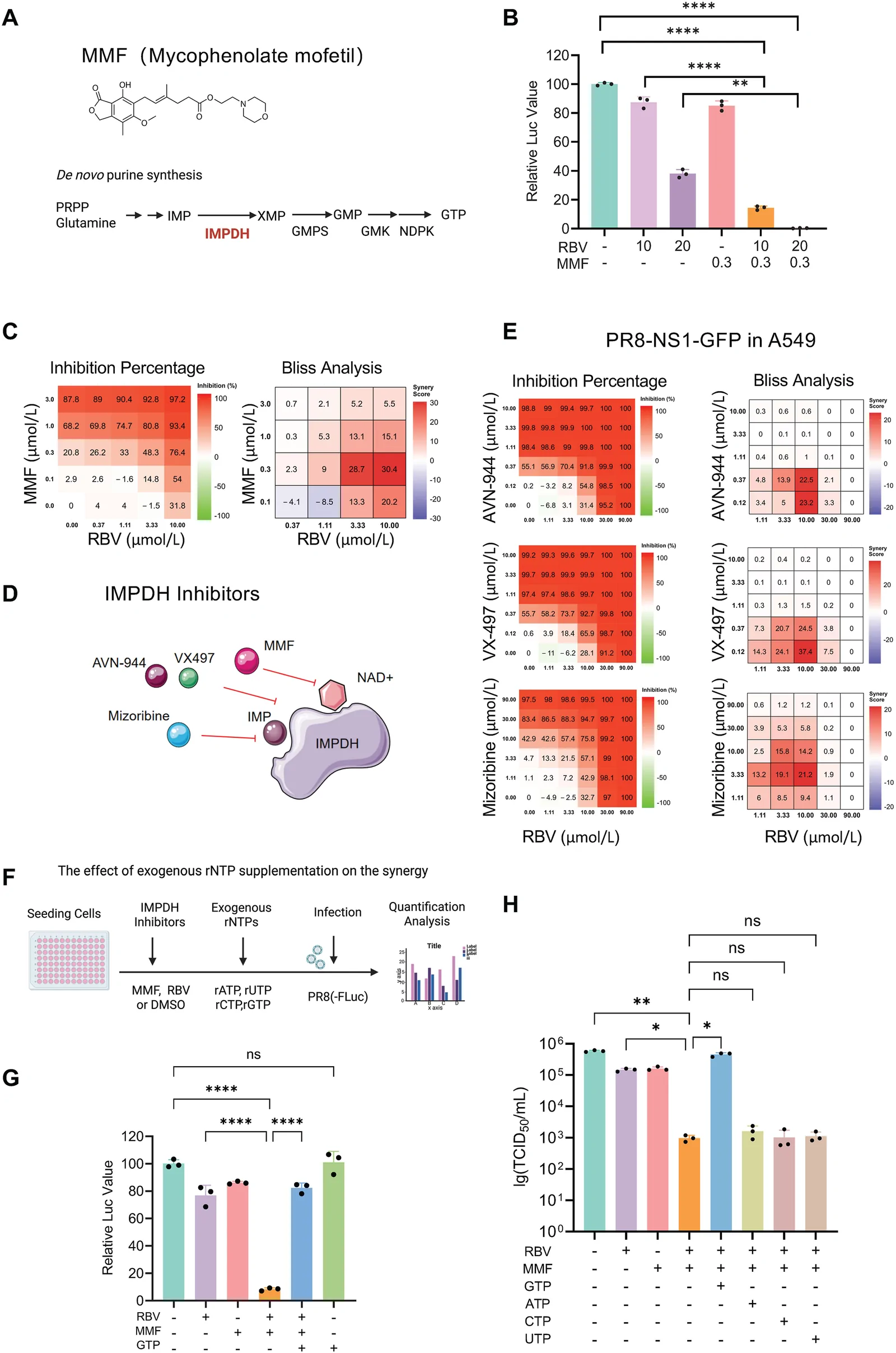
MMF and other IMPDH inhibitors synergize with ribavirin by depleting cellular GTP pool. (A) The chemical structure of MMF and schematic diagram of the *de novo* purine synthesis. (B) The synergistic effect of RBV with MMF in A549 cells. A549 cells were treated with the indicated drugs and infected with PR8-NS1-Fluc. Luciferase activity was quantified at 24 h post-infection. Data are presented as mean ± SD (*n* = 3 independent biological replicates). ∗∗*P* < 0.01, ∗∗∗∗*P* < 0.0001, one-way analysis of variance (ANOVA) with Tukey’s multiple comparison test. (C) The synergistic effect of MMF with RBV in Huh 7 cells. The inhibition percentage and synergy score matrix for RBV and MMF against PR8-NS1-Fluc under various concentration combinations were evaluated. Luciferase activity was quantified at 24 h post-infection. (D) Schematic illustration of IMPDH inhibitors targeting IMPDH. (E) The synergistic effect of other IMPDH inhibitors with RBV against PR8-NS1-GFP in Huh7 cells. The inhibition percentage and synergy score matrix for different IMPDH inhibitors against PR8-NS1-GFP were assessed. Huh7 cells were treated with the indicated drugs and followed by PR8-NS1-GFP infection, cells were fixed with paraformaldehyde and stained with DAPI after 24 h post-infection. Then the cells were analyzed by high-content screen system. The inhibition percentage and synergy score matrix for RBV and individual IMPDH inhibitors against PR8-NS1-GFP in various concentration combinations were evaluated. (F) Schematic representation showing the workflow of the exogenous NTP supplementation assay. (G) The exogenous GTP supplementation reversed the synergy between RBV and MMF by PR8-NS1-FLuc. A549 cells were treated with the indicated drugs (6 groups): DMSO (vehicle control), RBV (10 μmol/L), MMF (0.3 μmol/L), RBV + MMF (10 and 0.3 μmol/L, respectively), RBV + MMF + GTP (10, 0.3, and 100 μmol/L, respectively), or GTP alone (100 μmol/L). Following treatment for 6 h, cells were infected with PR8-NS1-FLuc at an MOI of 0.5 in the presence of the inhibitors. At 36 h post-infection, FLuc luminescence was measured, and the extent of inhibition was determined for each treatment group. Data are presented as mean ± SD (*n* = 3 independent biological replicates). ∗∗∗∗*P* < 0.0001, ns *P* > 0.05, one way ANOVA with Tukey’s multiple comparison test. (H) The exogenous GTP supplementation reversed the synergy between RBV and MMF by WT PR8. A549 cells were treated with the indicated drugs, followed by treatment with 100 μmol/L rNTPs. After a 6-h incubation, cells were infected with PR8-WT at an MOI of 0.5. Viral titers in the culture supernatants of each treatment group were measured at 36 h post-infection. Data are presented as mean ± SD (*n* = 3 independent biological replicates). ∗*P* < 0.05, ∗∗*P* < 0.01, ns *P* > 0.05, one-way ANOVA with Tukey’s multiple comparison test.

IMPDH is required for *de novo* guanosine biosynthesis^32^, ^33^, ^34^. Inhibition of IMPDH reduces the conversion of IMP to XMP, thereby decreasing intracellular GTP pools without significantly impacting ATP or other pyrimidine nucleotides (CTP and UTP) synthesis (Fig. 2A). Therefore, we aimed to investigate whether the reduction of GTP might contribute to the synergy between RBV and IMPDH inhibitors against influenza virus replication. As illustrated in Fig. 2F, exogenous nucleoside triphosphates (ATP, GTP, CTP or UTP) were supplemented to determine whether the synergistic antiviral effect of IMPDH inhibitor with RBV could be reversed. The results showed that GTP supplementation could reverse the synergy between MMF and RBV against IAV infection, whereas GTP alone didn’t alter viral infection through luciferase-based assays (Fig. 2G). Similarly, we also qualified viral titers of WT PR8 strain and reconfirmed exogenous supplementation of GTP could rescue the inhibition of IMPDH and thus reverse the synergy with RBV (Fig. 2H). It suggests that the depletion of GTP pool is essential for this synergy, although direct measurement of GTP levels would be required to fully confirm this mechanism. Taken together, these results demonstrate that MMF as well as other IMPDH inhibitors synergize with RBV against IAV through depleting intracellular GTP pools.

### 3.3 Mycophenolate mofetil exhibited synergistic effect with ribavirin by targeting host IMPDH2

We further validated whether IMPDH is the host target of MMF and contributes to the synergistic effect with RBV. IMPDH family has two members, IMPDH1 and IMPDH2^35^, both are capable to catalyze the nicotinamide adenine dinucleotide (NAD+)-dependent oxidation of IMP to XMP, which is the key step in *de novo* synthesis of GTP. In the tested cell lines A549 and Huh 7, IMPDH1 showed much lower levels compared with IMPDH2. In addition, knocking out IMPDH1 led to cell death and may interfere the following validations. Therefore, we knocked down IMPDH2 by transducing lentivirus with targeting shRNA and prepared the stable cell line by puromycin selection. The knockdown efficiency of the two shRNAs was validated by Western blot and quantitative PCR in both protein and mRNA levels (Supporting Information Fig. S3A‒C), respectively.

Next, we treated shRNA-transduced A549 cell lines with DMSO, RBV, RBV+GTP, or GTP alone, followed by infection with PR8-NS1-GFP. The results demonstrated that IMPDH2 knockdown alone did not significantly inhibit influenza virus replication but markedly enhanced the antiviral efficacy of RBV, indicating a robust synergy between IMPDH2 knockdown and RBV treatment. Additionally, exogenous GTP supplementation reversed this synergy, whereas GTP alone had no effect on PR8 virus replication (Fig. 3A). Furthermore, we confirmed that only GTP, and not ATP (another purine nucleotide), could reverse the synergy between IMPDH2 knockdown and RBV treatment (Fig. S3D). The reporter virus, PR8-NS1-FLuc, was also utilized to reconfirm the mechanism of this synergy. We similarly found that knocking down IMPDH2 increased the inhibition percentage of RBV from ∼53.4% to ∼92.2% (Fig. 3B). Additionally, viral titration assay utilizing WT PR8 also confirmed that down-regulation of IMPDH2 could synergize with RBV to inhibit PR8 replication (Fig. 3C). Consistent with these observations, only GTP—and not ATP, CTP, or UTP—reversed the synergistic effect (Fig. 3D), underscoring the essential role of IMPDH-mediated *de novo* GTP synthesis in the synergy between MMF and RBV.

**Figure 3:**
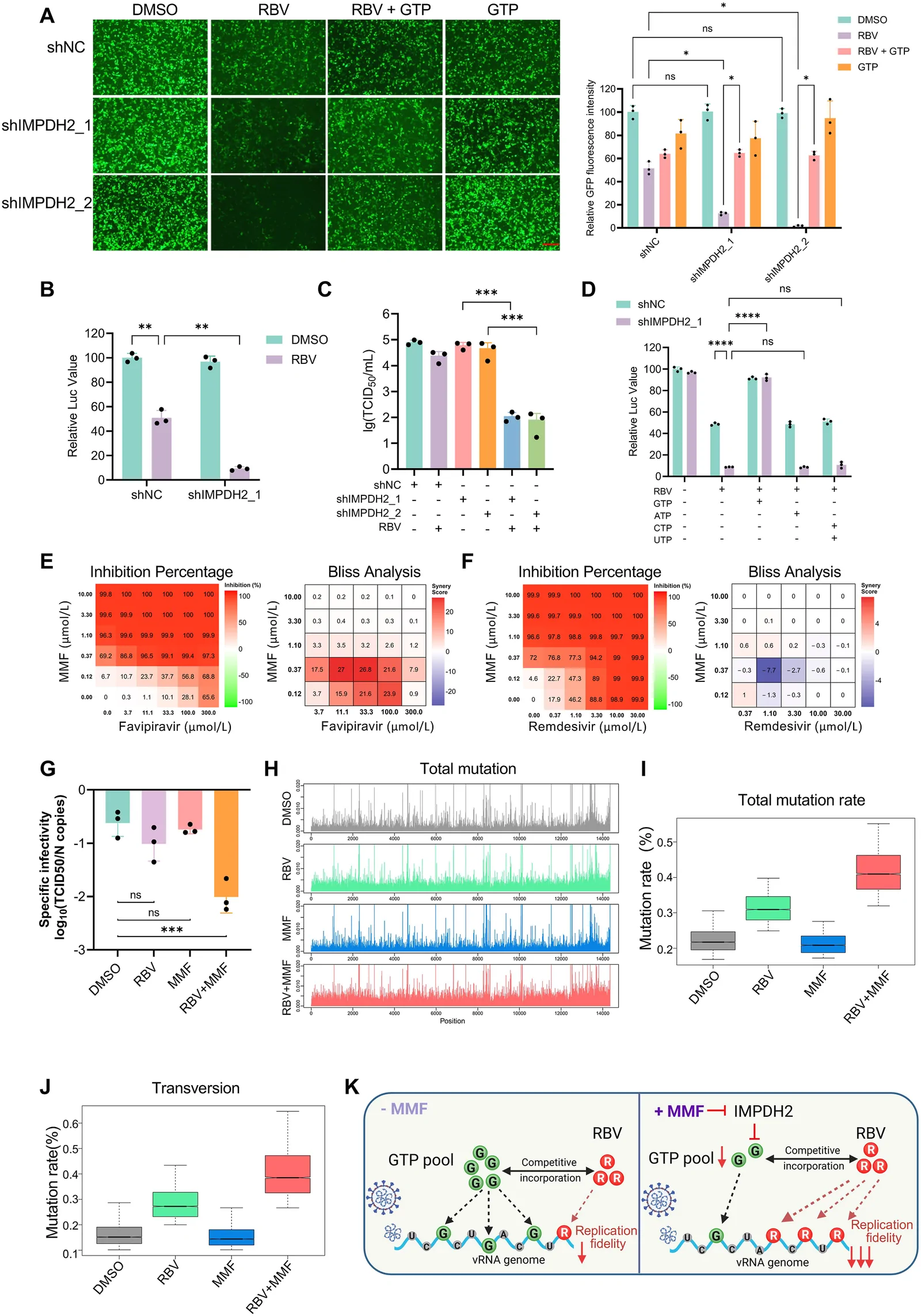
MMF showed synergy with ribavirin by targeting host IMPDH2. (A) Exogenous supplementation of GTP reversed the synergistic antiviral effect of RBV with IMPDH2 knockdown. shNC or shIMPDH2_1 transduced A549 cells were treated with or without RBV at the concentration of 20 μmol/L for 24 h, followed by treatment with or without 100 μmol/L GTP. Then GFP fluorescence was qualified after 24 h infection with PR8-NS1-GFP at an MOI of 0.5. The bar chart on the right shows the relative GFP fluorescence intensity in each group. Data are presented as mean ± SD (*n* = 3 independent biological replicates). ∗*P* < 0.05, ns *P* > 0.05, one-way ANOVA with Tukey’s multiple comparison test. (B) Luciferase assay was employed to evaluate the synergistic antiviral effect of IMPDH2 knockdown with RBV. A549 cells were transduced with shNC or shIMPDH2_1, subsequently treated with 20 μmol/L RBV or DMSO, and infected with PR8-NS1-Fluc at an MOI of 0.5 for 24 h. The luciferase activity in cell lysates was then measured to quantify viral infection. Data are presented as mean ± SD (*n* = 3 independent biological replicates). ∗∗*P* < 0.01, one-way ANOVA with Tukey’s multiple comparison test. (C) Exogenous supplementation of GTP reversed the synergistic antiviral effect by WT PR8 infection. A549 cells were transduced with shNC or shIMPDH2, subsequently treated with 20 μmol/L RBV or DMSO, followed by infection with WT PR8. Viral titers in the culture supernatants of each treatment group were measured at 36 h post-infection. Data are presented as mean ± SD (*n* = 3 independent biological replicates). ∗∗∗*P* < 0.001, one-way ANOVA with Tukey’s multiple comparison test. (D) Exogenous supplementation of GTP reversed the synergistic antiviral effect by PR8-NS1-Fluc infection. The shNC or shIMPDH2 transduced A549 cell lines were treated with 20 μmol/L RBV and subsequently supplemented with rNTPs at 100 μmol/L. Following a 6-h incubation, cells were infected with PR8-NS1-FLuc at an MOI of 0.5. Luciferase activity was measured at 36 h post-infection to determine the inhibition percentage of each treatment group. Data are presented as mean ± SD (*n* = 3 independent biological replicates). ∗∗∗∗*P* < 0.0001, ns *P* > 0.05, one-way ANOVA with Tukey’s multiple comparison test. (E) The synergistic effect of favipiravir with MMF against PR8-NS1-GFP in Huh 7 cells. The inhibition percentage and synergy score matrix for favipiravir and MMF against PR8-NS1-GFP were assessed. Huh7 cells were treated with the indicated drugs and followed by PR8-NS1-GFP infection, cells were fixed with paraformaldehyde and stained with DAPI after 24 h post-infection. Then the cells were analyzed by high-content screen system. The inhibition percentage and synergy score matrix for favipiravir and MMF against PR8-NS1-GFP under various concentration combinations were evaluated. (F) The synergistic effect of remdesivir with MMF against PR8-NS1-GFP in Huh7 cells. The inhibition percentage and synergy score matrix for remdesivir and MMF against PR8-NS1-GFP were assessed. Huh7 cells were treated with the indicated drugs and followed by PR8-NS1-GFP infection, cells were fixed with paraformaldehyde and stained with DAPI after 24 h post-infection. Then the cells were analyzed by high-content screen system. The inhibition percentage and synergy score matrix for remdesivir and MMF against PR8-NS1-GFP under various concentration combinations were evaluated. (G) Specific infectivity of PR8 virus in the presence of various inhibitors. Huh7 cells were treated with DMSO, RBV (10 μmol/L), MMF (0.1 μmol/L), or RBV + MMF, followed by infection with PR8 virus at an MOI of 0.03. Viral supernatants were harvested 24 h post-infection, the viral supernatant was harvested and subjected to serial passaging in the presence of the respective drugs. The supernatant from the second passage (P2) was collected. Viral titer was determined by the TCID_50_ assay, while the viral genome copy number was quantified by qPCR. Data are presented as mean ± SD (*n* = 3 independent biological replicates). ∗∗∗*P* < 0.001, ns *P* > 0.05, one-way ANOVA with Tukey’s multiple comparison test. (H) Total mutation rate per site separately in PR8 vRNA. Position-wise mutation density profiles across the viral genome (0–14,000 nucleotides) for each treatment group: DMSO, RBV, MMF, and RBV + MMF. Each subplot displays the per-site mutation frequency along the entire genome. The RBV + MMF group represents combined treatment with RBV and MMF. (I) Total mutation rate in PR8 vRNA. Box plots showing the total mutation rate in the RNA viral genome across four treatment groups: DMSO, RBV, MMF, and RBV + MMF. (J) Transition frequency of C-to-U and G-to-A in PR8 vRNA. Box plots showing the frequency of transition mutations in the RNA viral genome across four treatment groups: DMSO, RBV, MMF, and RBV + MMF. (K) Schematic illustration of the potential broad-spectrum synergistic mechanism between MMF and RBV against RNA viruses. Ribavirin is metabolized intracellularly to ribavirin triphosphate (RTP), which competes with GTP for incorporation into the newly synthesized viral RNA genome. This competition leads to a reduction in replication fidelity. When ribavirin is used in combination with MMF, the resulting decrease in intracellular GTP levels enhances the misincorporation of ribavirin into the viral RNA genome, thereby further impairing viral replication.

### 3.4 Mycophenolate mofetil synergizes with guanosine analogues by promoting their misincorporation into the viral RNA genome

RBV is intracellularly metabolized into ribavirin monophosphate (RBV-MP) and subsequently into ribavirin triphosphate (RBV-TP)^36^. These metabolites mediate distinct antiviral mechanisms: RBV-TP, acting as a nucleoside analogue, incorporates into the viral genome to induce lethal mutagenesis; While RBV-MP inhibits IMPDH, thereby reducing intracellular GTP levels to suppress viral replication. To investigate whether the observed synergy is primarily due to increased misincorporation mechanism of RBV or inhibition of cellular IMPDH, we provided multiple lines of evidence. First, to further confirm that the guanosine analogue activity of ribavirin is primarily responsible for its synergy with MMF, we evaluated favipiravir, another guanosine analogue that resembles ribavirin but lacks IMPDH inhibitory activity. The combination of MMF and favipiravir exhibited strong synergistic antiviral activity against IAV (Fig. 3E). In contrast, no such synergy was observed with the non-guanosine analogue remdesivir (Fig. 3F). These results indicate that the guanosine analogue activity is the key determinant for synergy with MMF. Second, to exclude the possibility that the synergy with MMF comes from the IMPDH inhibitory activity of ribavirin, we evaluated mizoribine, an IMPDH specific inhibitor without nucleotide analogue activity. The results showed these combinations did not exhibit significant synergistic antiviral effects (Fig. S3E), indicating that simple additive or synergistic inhibition at the IMPDH enzyme level is insufficient to explain the potent synergy observed with MMF and ribavirin. To further validate whether MMF exerts its synergistic effect by enhancing the impact of RBV-TP on viral genome mutagenesis, we first measured specific infectivity (TCID_50_/genome copy), a hallmark of lethal mutagenesis^37^. Compared to the DMSO control, serial passaging of PR8-NS1-GFP with RBV treatment resulted in a more than 2-fold reduction in specific infectivity, whereas MMF treatment alone showed no significant effect. In contrast, the combination of RBV and MMF led to a dramatic reduction of over 20-fold (Fig. 3G). This phenotype aligns with the mutagenic mechanism of RBV-TP and suggests that MMF enhances the mutagenic activity of RBV to achieve synergy. To directly validate this mechanism, we performed viral genome sequencing to quantitatively analyze the mutation frequency of PR8 after three consecutive passages under drug pressure. Compared to the DMSO group, the total mutation frequency of the viral genome significantly increased with RBV treatment, whereas MMF alone showed no significant effect. Furthermore, MMF potentiated the RBV-induced increase in genomic total mutation frequency (Fig. 3H and I). This mutagenesis was characterized primarily by a rise in C-to-U and G-to-A transitions (Fig. 3J), rather than an increase in C-to-G or G-to-C transversions (Fig. S3F), which aligns precisely with the mutational signature of RBV-TP incorporation into the viral RNA genome. These results further demonstrate that MMF, by suppressing cellular GTP levels, promotes the increased misincorporation of RBV-TP into the viral RNA genome, thereby inhibiting viral replication (Fig. 3K).

### 3.5 The synergistic antiviral activity of IMPDH inhibitors with ribavirin applies to other RNA viruses

We have confirmed that MMF synergizes with RBV against influenza virus by depleting host cell GTP pools. Given that RBV functions as a competitive nucleoside analogue of GTP, MMF may deplete intracellular GTP pool and therefore increase RBV incorporation efficiency and leading to stronger inhibitory potency. This synergy mechanism is not specific to influenza virus and may also apply to other RNA viruses. Therefore, we tried to determine whether the MMF-RBV synergistic effect extends to other RNA viruses (Fig. 4A).

**Figure 4:**
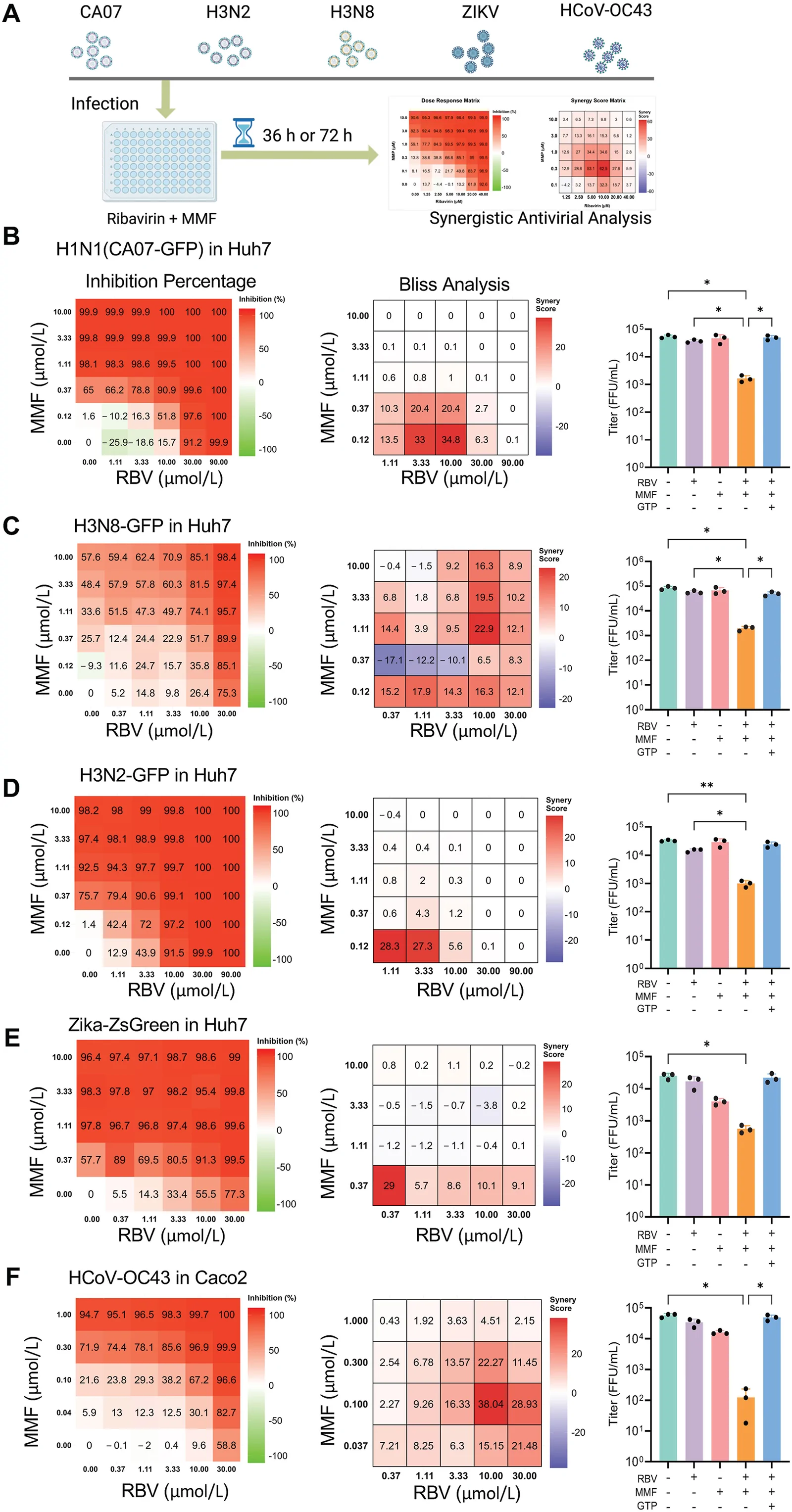
MMF showed synergistic effects with ribavirin against other RNA viruses. (A) Schematic representation showing the workflow to demonstrate the synergistic effect between MMF and RBV against other RNA viruses. (B) Synergistic effect of MMF (0.37 μmol/L) in combination with RBV (10 μmol/L) against H1N1 CA07-GFP in Huh 7 cell line. Huh7 cells were treated with RBV in combination with MMF under indicated concentrations. The inhibition percentages and synergy scores were determined by quantifying the GFP fluorescence after 36 h infection. For the titer quantification assay, another treatment group (RBV + MMF + GTP) was included. The viral titers in the cell culture supernatants were quantified at 48 h post-infection. Data are presented as mean ± SD (*n* = 3 independent biological replicates). ∗*P* < 0.05, one-way ANOVA with Tukey’s multiple comparison test. (C) Synergistic effect of MMF (1.11 μmol/L) in combination with RBV (10 μmol/L) against H3N8-GFP in Huh7 cell line. Huh7 cells were treated with RBV in combination with MMF under indicated concentrations. The inhibition percentages and synergy scores were determined by quantifying the GFP fluorescence after 36 h infection. For the titer quantification assay, another treatment group (RBV + MMF + GTP) was included. The viral titers in the cell culture supernatants were quantified at 48 h post-infection. Data are presented as mean ± SD (*n* = 3 independent biological replicates). ∗*P* < 0.05, one-way ANOVA with Tukey’s multiple comparison test. (D) Synergistic effect of MMF (0.12 μmol/L) in combination with RBV (3.33 μmol/L) against H3N2-GFP in Huh7 cell line. Huh7 cells were treated with RBV in combination with MMF under indicated concentrations. The inhibition percentages and synergy scores were determined by quantifying the GFP fluorescence after 36 h infection. For the titer quantification assay, another treatment group (RBV + MMF + GTP) was included. The viral titers in the cell culture supernatants were quantified at 48 h post-infection. Data are presented as mean ± SD (*n* = 3 independent biological replicates). ∗*P* < 0.05, ∗∗*P* < 0.01, one-way ANOVA with Tukey’s multiple comparison test. (E) Synergistic effect of MMF (0.37 μmol/L) in combination with RBV (10 μmol/L) against ZIKV-ZsGreen in Huh7 cell line. Huh7 cells were treated with RBV in combination with MMF under indicated concentrations. The inhibition percentages and synergy scores were determined by quantifying the ZsGreen fluorescence after 36 h infection. For the titer quantification assay, another treatment group (RBV + MMF + GTP) was included. The viral titers in the cell culture supernatants were quantified at 48 h post-infection. Data are presented as mean ± SD (*n* = 3 independent biological replicates. ∗*P* < 0.05, one-way ANOVA with Tukey’s multiple comparison test. (F) Synergistic effect of MMF (0.1 μmol/L) in combination with RBV (10 μmol/L) against HCoV-OC43 in Caco 2 cell line. Caco2 cells were treated with RBV in combination with MMF under indicated concentrations. The inhibition percentages and synergy scores were determined by quantifying the NP-staining fluorescence after 72 h infection. For the titer quantification assay, another treatment group (RBV + MMF + GTP) was included. The viral titers in the cell culture supernatants were quantified at 72 h post-infection. Data are presented as mean ± SD (*n* = 3 independent biological replicates). ∗*P* < 0.05, one-way ANOVA with Tukey’s multiple comparison test.

Firstly, we tested this effect using another H1N1 strain CA07 (A/California/08/2009) in Huh 7 cells. Based on the GFP quantification assay, we performed 5 × 5 dose-response analysis. This synergy was observed in the submicromolar range of MMF with RBV (Fig. 4B, Supporting Information Fig. S4A). Consistent with MMF, other two IMPDH inhibitors (AVN-944 and mizoribine) also showed synergy with RBV (Fig. S4B). Expanding to other influenza subtypes, such as H3N2 (A/China/LZP/2017), a currently circulating and seasonal influenza strain, and H3N8 (A/China/ZMD-22-2/2022), we also observed similar synergistic antiviral activity between MMF and RBV (Fig. 4C and D, and Supporting Information Fig. S5A and B), although the synergy was less pronounced for H3N2 compared to other IAV strains. Furthermore, it also confirmed this effect could be reversed by supplementing exogenous GTP (Fig. 4B‒D). The results showed that this synergy is conserved against influenza virus.

We further extended our investigation to other RNA virus families. HCoV-OC43 is a circulating human coronavirus, while Zika virus belongs to the family of Flaviviridae; both contain a single-stranded, positive-sense RNA genome. We aimed to determine whether the combination of MMF and RBV also exhibits synergistic effects in HCoV-OC43 and ZIKV infection models (Fig. 4E and F, and Supporting Information Fig. S6A and B). Through a 4 × 5 dose-response matrix analysis, we confirmed that MMF synergizes with RBV against these two RNA viruses, although the synergy was less pronounced for ZIKV. A viral titration assay further supported this conclusion, confirming that the depletion of the cellular GTP pool was probably the mechanism through which MMF exerted its synergistic effect. Collectively, our findings demonstrate that the synergistic antiviral activity of IMPDH inhibitors with RBV is not restricted to influenza virus but extends to other RNA viruses as well.

### 3.6 The synergistic antiviral effects of mycophenolate mofetil with ribavirin against influenza virus in a mouse model

We observed synergistic antiviral activity between MMF and RBV across a range of RNA viruses *in vitro*. Therefore, we sought to determine whether this combination would also exhibit synergistic effects *in vivo*. To evaluate the antiviral efficacy of the drug combination, a mouse infection model was utilized, in which 6- to 8-week-old BALB/c mice were intranasally inoculated with the PR8 virus and administered the drugs *via* oral gavage twice daily (Fig. 5A).

**Figure 5:**
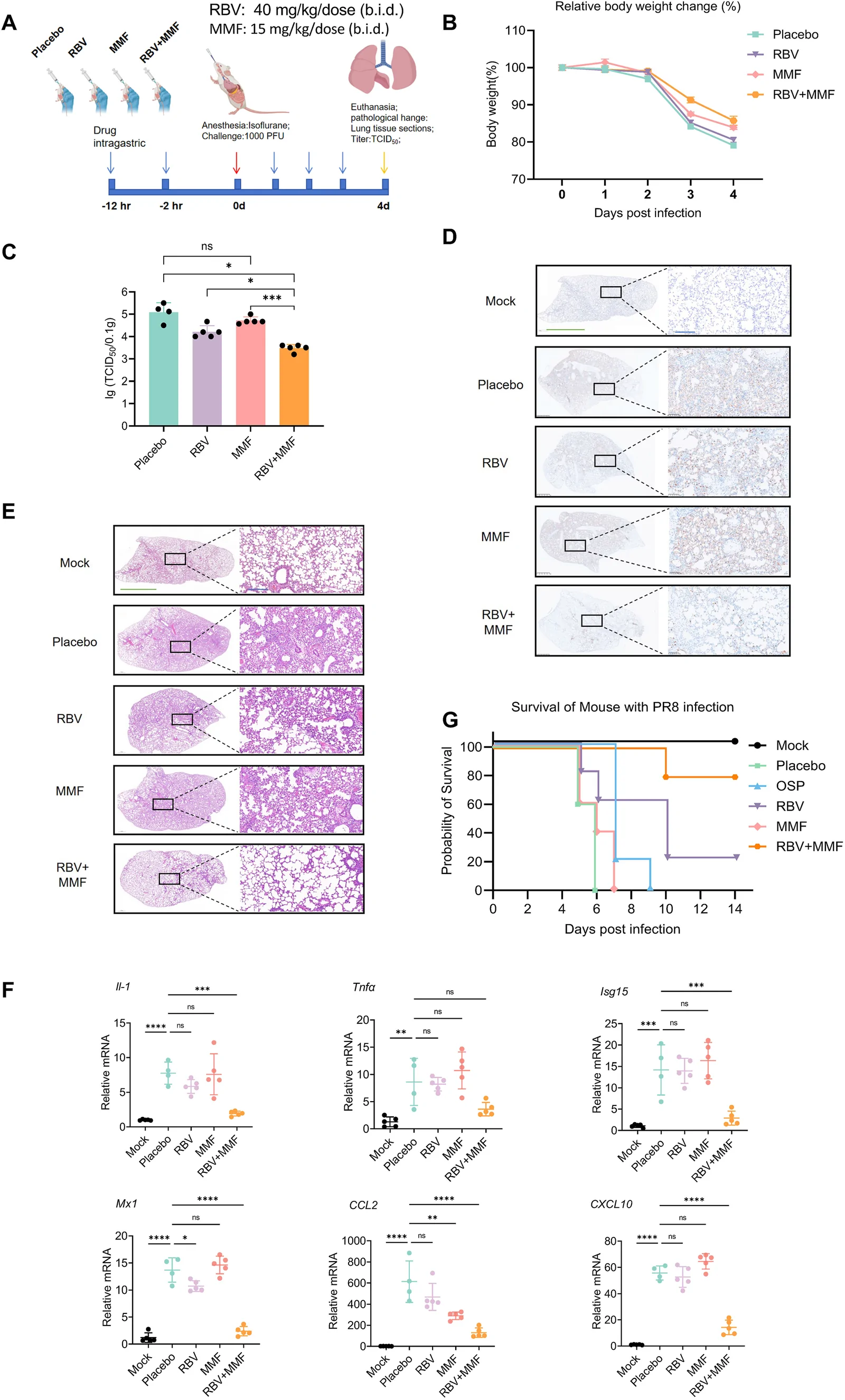
Synergistic antiviral effects of ribavirin and MMF *in vivo.* (A) Female BALB/c mice (6‒8 weeks) were administrated with PBS (Placebo, *n* = 4), RBV (40 mg/kg, b.i.d., *n* = 5), MMF (15 mg/kg, b.i.d., *n* = 5), or the combination (RBV 40 mg/kg, b.i.d. + MMF 15 mg/kg, b.i.d., *n* = 5) *via* oral gavage 2 h prior to intranasal challenge with 1000 PFU PR8 (H1N1). (B) Weight changes in mice. The mice from each group were weighed every day after infection. Effect on body weight relative to that at Day 0 for all mice. Mean ± SD is shown. (C) Determination of viral load in lung tissue. Mouse Lung tissues were collected and homogenized for viral titer quantification at 4 days post-infection. Mean ± SD is shown. ∗*P* < 0.05, ∗∗∗*P* < 0.001, ns *P* > 0.05, using one-way ANOVA with Tukey’s multiple comparison test. (D) Immunohistochemical examination of lung tissue sections stained with influenza NP antibody. Mouse Lung tissues from mock and infected mice were collected and stained with PR8-NP antibody to monitor viral antigen at 4 days post-infection. Representative images are shown for each group. Scale bar, 3 mm (red), 100 μm (blue). (E) Histopathological examination of lung tissue sections stained with hematoxylin and eosin (H&E). Mouse lung tissues from mock and infected mice were collected and stained with HE at 4 days post-infection. Representative images are shown for each group. Scale bar, 3 mm (red), 100 μm (blue). (F) Quantitative qPCR analysis of cytokines expression in lung tissue. Mouse lung tissues were collected and lysed in TRIzol reagent at 4 days post-infection. Following total RNA extraction, qPCR was performed to quantify the mRNA expression levels of the indicated cytokines. Mean ± SD is shown. (G) Fourteen-day *in vivo* evaluation of the antiviral efficacy of combined RBV and MMF treatment in a mose model. Female BALB/c mice (6‒8 weeks) were administrated with PBS (Placebo, *n* = 4), oseltamivir phosphate (OSP, 10 mg/kg, b.i.d., *n* = 5), RBV (40 mg/kg, b.i.d., *n* = 5), MMF (15 mg/kg, b.i.d., *n* = 5), or the combination (RBV 40 mg/kg b.i.d. + MMF 15 mg/kg b.i.d., *n* = 5) *via* oral gavage 2 h prior to intranasal challenge with 1000 PFU PR8 (H1N1). Mice were monitored daily for survival over a 14-day period post-infection.

Based on a systematic review of previous reports, we selected dose ranges of 40–160 mg/kg/day for RBV^38^, ^39^, ^40^, ^41^, ^42^ and 30–60 mg/kg/day for MMF^43^, ^44^, ^45^ for *in vivo* evaluation in mice. Due to the saturable oral absorption and short half-lives of both drugs, we adopted a twice-daily (b.i.d.) dosing regimen to maintain stable drug levels^46^^,^^47^. In a preliminary dose-ranging experiment (Supporting Information Fig. S7A), RBV at 40 and 80 mg/kg/dose b.i.d. (equivalent to 80 and 160 mg/kg/day) showed moderate antiviral activity, whereas 20 mg/kg/dose b.i.d. was ineffective. For MMF, no significant single-agent antiviral effect was observed even at doses reported to be immunosuppressive. Therefore, we selected 40 mg/kg/dose b.i.d. for RBV and 15 mg/kg/dose b.i.d. for MMF as starting doses for subsequent combination studies.

In this model, persistent weight loss was observed (Fig. 5B), along with robust viral replication at 4 days post-infection, as determined by high viral titers in the lungs (Fig. 5C) and confirmed by immunohistochemical detection of the viral NP antigen (Fig. 5D) and NP mRNA (Fig. S7B). Pathological examination revealed shedding of infected epithelial cells in bronchioles, extensive alveolar atrophy, thickening of alveolar walls, and significant inflammatory cell infiltration, as validated by histological analysis (Fig. 5E). Furthermore, a pronounced inflammatory response was evident, supported by elevated transcriptional levels of inflammatory factors measured *via* qPCR (Fig. 5F).

When animals were administered with RBV alone at a dose of 40 mg/kg, lung pathology was only mildly alleviated, with prominent epithelial cell infection and inflammatory cell infiltration still evident. Minimal reductions in viral titers and slight improvements in pathological conditions were observed. Similarly, mice treated with MMF alone at a dose of 15 mg/kg exhibited no significant changes in viral titers or lung pathology 4 days post-infection. Notably, the concurrent administration of RBV and MMF resulted in a marked reduction in viral titers—approximately 10-fold lower than that achieved with RBV monotherapy (Fig. 5C). Furthermore, the combined treatment significantly protected lung architecture, maintaining intact bronchiolar and alveolar structures with clear contours (Fig. 5D and E). And the induction of inflammatory factors was also alleviated by the combined treatment (Fig. 5F). Over an extended observation period lasting up to 14 days post-infection, both body weight changes and mortality were closely monitored (Fig. 5G, Fig. S7C). In the placebo group, mice exhibited rapid weight loss, and mortality reached 100% at Day 6 post-infection. RBV monotherapy provided modest protection, with substantial weight loss and a 20% survival rate by Day 14. MMF monotherapy conferred no obvious benefit, as all mice in this group succumbed to infection by Day 7 post-infection. In striking contrast, the RBV and MMF combination exhibited substantially enhanced antiviral efficacy: 80% of mice survived to the end of the observation period and showed significantly less body weight loss than the placebo group. Moreover, mice treated with the combination began regaining weight rapidly from Day 9 post-infection. A group treated with oseltamivir phosphate as a positive control, the treatment of oseltamivir delayed the mortality as expected. Collectively, the combination of RBV and MMF conferred superior protection against lethality and promoted faster body weight recovery relative to either monotherapy or placebo treatment.

## 4 Discussion

Emerging and re-emerging viruses continue to threaten human society. Broad-spectrum antiviral drugs represent one of the most promising strategies to combat these threats. For instance, nucleoside analogues inhibit viral replication by interfering with viral RNA-dependent RNA polymerase. To further maximum the antiviral potential of such inhibitors, combinations of multiple direct-acting antiviral agents are commonly employed. A well-known example is Epclusa^48^, ^49^, ^50^, a medication for hepatitis C virus (HCV) that combines sofosbuvir^48^^,^^51^^,^^52^ and velpatasvir, which target the viral proteins NS5B and NS5A, respectively. The conventional principle of combination therapy is that each component must possess direct antiviral activity. An advantage of this approach is that it typically results in additive antiviral effects, and in some cases, synergistic interactions may occur, producing beneficial outcomes that surpass simple additivity. However, a major limitation is that both components must exhibit antiviral activity, which significantly restricts the number of possible combinations, especially given the limited availability of approved direct-acting antiviral drugs, particularly for certain pathogens. In contrast, there exists a relatively large number of drugs targeting host cellular pathways. This raises an important question: can non-antiviral agents that target distinct cellular pathways synergize with a known antiviral drug? Indeed, this strategy has been widely explored in cancer therapy. For example, stiripentol, an inhibitor of lactate dehydrogenase A, lacks intrinsic anti-tumor activity but significantly enhances the efficacy of platinum-based chemotherapy^53^. Similarly, in the field of antiviral therapy, Paxlovid serves as a well-established example: its component ritonavir does not exhibit anti-coronavirus activity but enhances the potency of nirmatrelvir by prolonging its half-life in humans. Another study reporting that two compounds identified by deep learning approaches potentiate with remdesivir despite lacking intrinsic antiviral activity^3^. Meanwhile, we also observed a substantial proportion of prior study first identifies compounds with direct antiviral activity and subsequently tests them for synergy. A representative example is the work by Schultz et al.^23^, which screened for single-agent antiviral compounds, identified pyrimidine biosynthesis inhibitors, and later demonstrated their synergy with pyrimidine analogues. Therefore, the new combination principle has the potential to overcome this limitation. It is worthwhile to investigate whether a specific antiviral drug can achieve synergy when combined with a non-antiviral inhibitor or low-potency antiviral inhibitors, and to elucidate the underlying mechanism of this synergy. Such research would expand the current scope of synergistic combinations and uncover novel mechanisms through which such synergy may occur.

Nucleoside analogues represent one of the most promising classes of antiviral agents, particularly due to their broad-spectrum activity. However, their efficacy varies across different viral families. Therefore, we aimed to investigate novel combinations that could enhance the antiviral potential of RBV. Such combinations would be especially valuable during the early stages of an emerging viral outbreak, when highly effective interventions are lacking. To date, few studies have reported on combinational antiviral drug screening. In response, we established a novel high-throughput synergistic screening system and screened over 3000 FDA-approved compounds to identify potential synergistic partners for RBV. Several compounds exhibited synergistic effects with RBV, among which MMF showed the highest potential. MMF is an immunosuppressive agent whose active metabolite is mycophenolic acid (MPA)^54^^,^^55^. Following oral administration, MMF is rapidly hydrolyzed *in vivo* to MPA, which exerts its immunosuppressive effects by selectively inhibiting the proliferation of T and B lymphocytes. This is achieved through the non-competitive inhibition of IMPDH, a key enzyme in the *de novo* synthesis pathway of guanosine nucleotides^34^^,^^56^^,^^57^. Clinically, MMF is used for the prevention of allograft rejection following renal, hepatic, and cardiac transplantation.

Further analysis confirmed that MMF significantly enhances the antiviral efficacy of RBV, even at concentrations where MMF alone exhibits minimal antiviral activity. Further mechanistic investigations demonstrated that MMF synergizes with RBV by targeting its cellular target, IMPDH. Inhibition of IMPDH reduces the conversion of IMP to XMP, thereby decreasing intracellular GTP pools. Since RBV is a competitive inhibitor of GTP, the reduction in cellular GTP potentially increases RBV incorporation into the viral genome, thereby disrupting replication fidelity and leading to stronger inhibitory potency^58^. This mechanism was reconfirmed by supplementation with exogenous GTP, which reversed the synergistic effect. We further validated this synergy in a mouse model, where MMF significantly augmented the antiviral potency of RBV against influenza virus *in vivo*, even though MMF alone exhibited very limited antiviral activity. These findings highlight the potential clinical application of MMF in combating RBV-treatable infectious diseases, especially those caused by unknown or emerging viruses.

Although previous work has described synergy between mycophenolic acid and RBV, the present study offers several substantial advances. First, we developed an unbiased high-throughput screening platform that identified multiple novel combination candidates from 3200 compounds and provided critical *in vivo* validation, demonstrating that MMF potentiates RBV even at doses lacking direct antiviral activity. Second, we elucidate a broad-spectrum mechanistic paradigm wherein MMF enhances RBV incorporation into viral genomes through intracellular GTP depletion—a mechanism that extends to other guanosine analogues, including favipiravir. Finally, we significantly expand the therapeutic scope by confirming this synergistic effect across multiple influenza subtypes and other phylogenetically distinct RNA viruses, such as coronavirus (HCoV-OC43) and Zika virus. Moreover, previous studies have also reported the synergistic inhibition of parainfluenza virus (PIV) by MPA and RBV^59^. Collectively, these findings establish GTP depletion as a broadly applicable and innovative therapeutic strategy with potential implications for combating diverse RNA viruses.

Interestingly, compared with the synergy of MMF depends on disrupting *de novo* purine synthesis, a recent study proved that interfering *de novo* pyrimidine by DHODH inhibitors significantly enhance the antiviral efficacy of a nucleoside analogue molnupiravir (a pyrimidine ribonucleoside analogue) against SARS-CoV-2, even though DHODH inhibitors alone do not show any anti-SARS-CoV-2 activity *in vivo*. Combining both studies, targeting host metabolism of nucleoside synthesis is a rational and general strategy to enhance the antiviral potential of broad antiviral nucleoside analogues. More importantly, we confirmed this synergy applies to multiple RNA virus families, highlighting this is a potential broad-spectrum synergy strategy. In summary, our work established a novel synergistic screening system and identifies new candidates that synergize with a nucleoside analogue, representing an unexplored strategy to develop innovative broad-spectrum antiviral interventions, especially in the response to emerging unknown viral disease.

## Author contributions

Qiangyun Ai, Huina Hu, Kaixiong Ma: writing, validation, investigation, formal analysis, data curation. Qingxing Wang: validation, investigation, formal analysis. Shenglin Huang, Yongzhi Lu: investigation. Nan Qi: resources, supervision. Jinsai Shang, Ruikun Du: resources, supervision, conceptualization. Shaobo Wang: supervision, writing, resources, project administration, formal analysis, data curation, conceptualization.

## Conflicts of interest

Shaobo Wang, Qiangyun Ai, Huina Hu, Kaixiong Ma, and Shenglin Huang are currently listed as inventors on a patent application that discloses the subject matter described in this paper.

## Appendix A Supporting information

The following is the Supporting Information to this article.
