# Supplementary material for: Identification of FDA-approved IMPDH inhibitors boosting the broad-spectrum antiviral potential of ribavirin through depleting cellular GTP pool
Source: Acta Pharm Sin B. 2026 May 20;16(10):6875–91. doi: 10.1016/j.apsb.2026.05.017 (PMC13712292; doi:10.1016/j.apsb.2026.05.017)
Supplement: Supplementary file 1 [file mmc1.pdf]

Supporting Information for

Original article

Identification of FDA-approved IMPDH inhibitors boosting the broad-spectrum antiviral potential of ribavirin through depleting cellular GTP pool

Qiangyun Ai[a,b,†], Huina Hu[b,c,†], Kaixiong Ma[a,b,†], Qingxing Wang[a,b], Shenglin Huang[b], Yongzhi Lu[a,b], Nan Qi[a,b], Jinsai Shang[a,b,*], Ruikun Du[d,*], Shaobo Wang[a,b,*]

[a]*State Key Laboratory of Respiratory Disease, The First Affiliated Hospital of Guangzhou Medical University, Guangzhou National Laboratory Clinical Base, School of Basic Medical Sciences, Guangzhou Medical University, Guangzhou 511436, China*

[b]*Guangzhou National Laboratory, Department of Basic Research, Guangzhou 510005, China*

[c]*Wuhan Institute of Virology, Chinese Academy of Sciences, Wuhan 430071, China; University of the Chinese Academy of Sciences, Beijing 100049, China*

[d]*Qingdao Academy of Chinese Medical Sciences, Shandong University of Traditional Chinese Medicine, Qingdao 266122, China*

Received 15 September 2025; received in revised form 27 March 2026; accepted 7 April 2026

[†]These authors made equal contributions to this work.

* Corresponding authors.

E-mail addresses: wang_shaobo@gzlab.ac.cn (Shaobo Wang); ruikun@sdutcm.edu.cn (Ruikun Du); shang_jinsai@gzlab.ac.cn (Jinsai Shang)

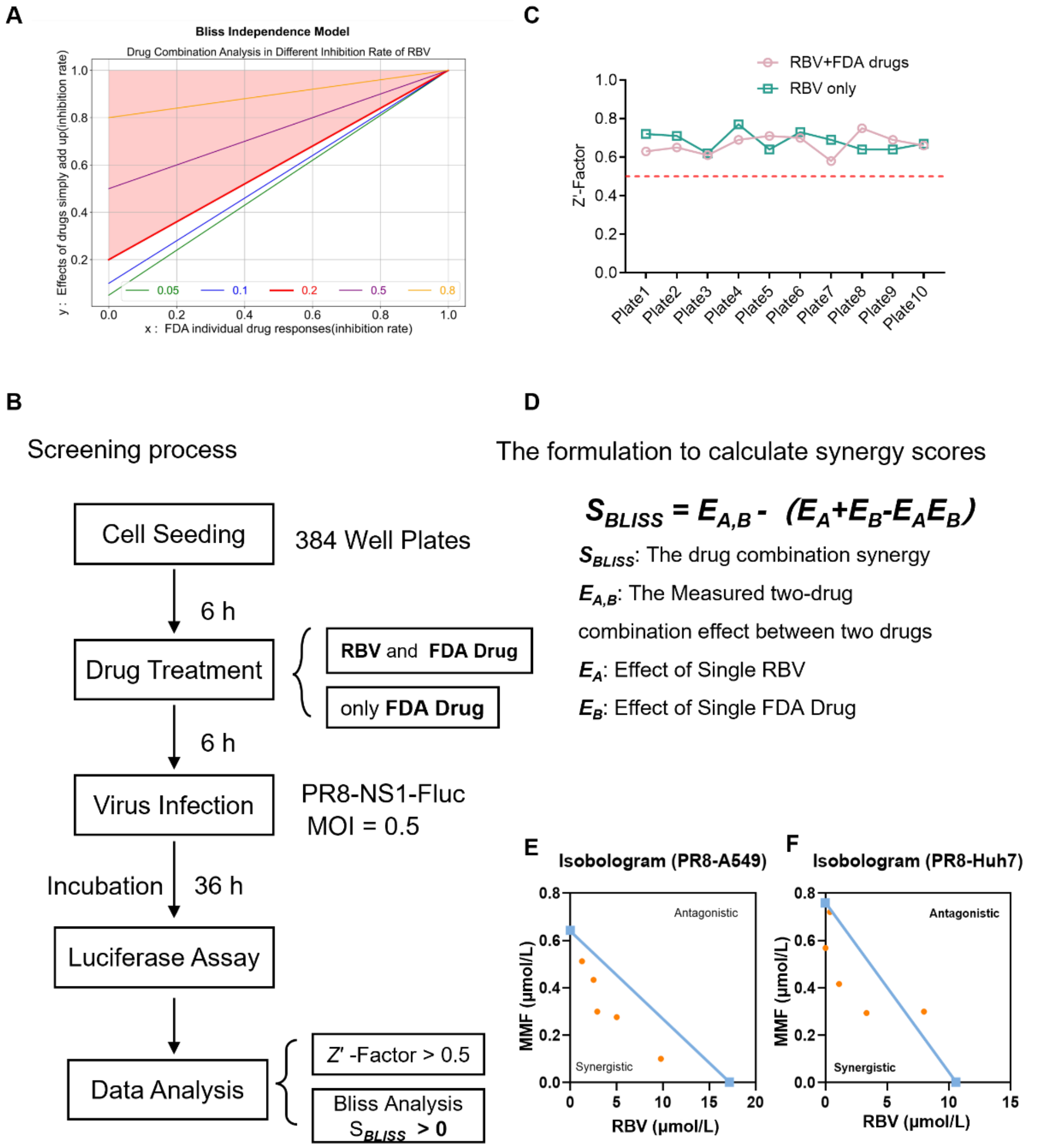

Figure S1. A high-throughput synergistic screening identified FDA-approved drugs. (A) RBV concentration selection for screening synergistic combinations in the Bliss Independent Analysis Framework. Bliss analysis windows for single-agent inhibition rates of RBV at different concentrations. The red-shaded region indicated the window when RBV alone achieves an inhibition percentage of more than 20%. (B) The workflow for the synergistic screening. *Z′*-factors of all plates for the synergistic combinations screening. A549 cells were seeded at 5,000 cells per well in 384-well black plates. After 6 h, RBV alone or the combinations of RBV with FDA-approved drugs (3,104 compounds) were added to the cell culture medium. Six hours later, cells were infected with PR8-NS1-FLuc at an MOI of 0.5 and incubated at 37 °C in a cell culture incubator. 36 h post-infection, luciferase assays were performed to measure FLuc luminescence values. *Z′*-factor and Bliss analysis for each plate was both conducted for further analysis. (C) *Z′*-Factor values for all 384-well plates in the high-throughput synergistic screening. The *Z′*-factor values were calculated based on the

negative and positive controls. (D) Bliss analysis calculation formula. The Bliss models quantify the degree of synergy excess over the multiplicative effect of single drugs as if they acted independently. S_{Bliss} denotes the synergy score. E_A or E_B denote the single-agent inhibition rates of RBV and FDA-approved drugs, respectively. $E_{A,\ B}$ is the antiviral inhibition rate of drug combinations measured in actual cell-based experiments. Concentration of RBV were selected based on pre-screening IC_{20} values to ensure the 20% inhibition ratio window aligns with Bliss synergy calculation requirements. (E) The synergistic interaction between MMF and RBV using isobologram analysis in A549 cells. The synergistic antiviral effects of the MMF–RBV combination against PR8 were quantitatively evaluated using isobologram analysis. Experimentally determined concentration pairs achieving 50% inhibition were then plotted on the same plot. Combination points falling on the line indicates an additive effect, while a point falling below the line indicates synergy. drug combination that fell within the synergistic region, with results presented in the figures. (F) The synergistic interaction between MMF and RBV using isobologram analysis in Huh7 cells. The synergistic antiviral effects of the MMF–RBV combination against PR8 in Huh7 cells were quantitatively evaluated using isobologram analysis. Experimentally determined concentration pairs achieving 50% inhibition were then plotted on the same plot.

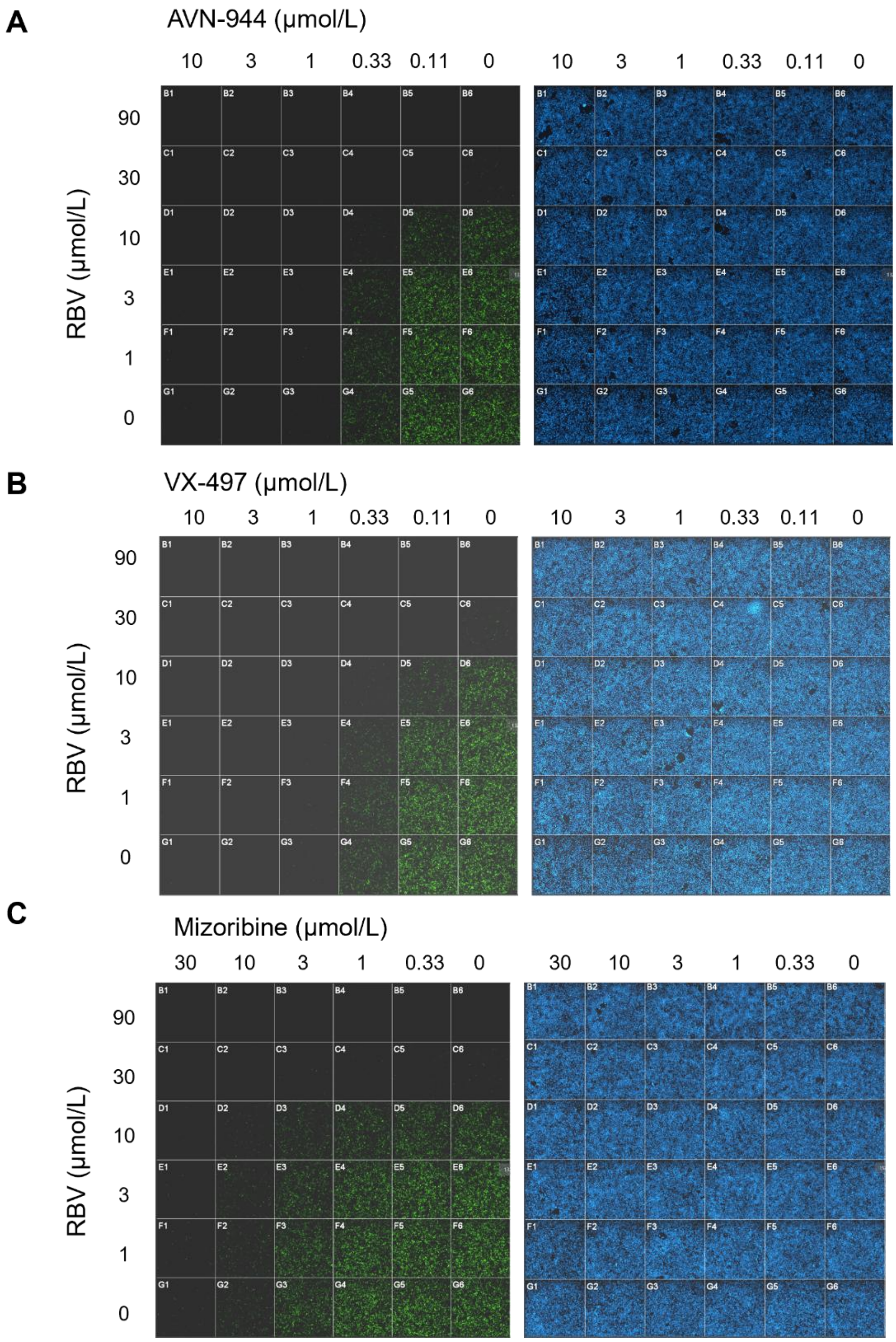

Figure S2. MMF and other IMPDH inhibitors can synergistic anti-influenza with ribavirin. (A) Synergistic effect of AVN-944 in combination with RBV against PR8-NS1-GFP in Huh7 cell line. Huh7 cells were treated with RBV in combination with AVN-944 in indicated concentrations. The inhibition percentages and synergy scores were determined by quantifying the GFP fluorescence after 24 h infection with PR8-NS1-GFP. Huh7 cells were fixed, stained with DAPI, and analyzed via high-content scanning. The left panel shows the images from high-content screening of GFP in the synergistic combination group, while the right panel displays DAPI staining. (B) Synergistic effect of VX-497 in combination with RBV against PR8-NS1-GFP in Huh7

cell line. Huh7 cells were treated with RBV in combination with VX-497 under indicated concentrations. The inhibition percentages and synergy scores were determined by quantifying the GFP fluorescence after 24 h infection with PR8-NS1-GFP. Huh7 cells were fixed, stained with DAPI, and analyzed via high-content scanning. The left panel shows the images from high-content screening of GFP in the synergistic combination group, while the right panel displays DAPI staining. (C) Synergistic effect of mizoribine in combination with RBV against PR8-NS1-GFP in Huh7 cell line. Huh7 cells were treated with RBV in combination with mizoribine under indicated concentrations. The inhibition percentages and synergy scores were determined by quantifying the GFP fluorescence after 24 h infection with PR8-NS1-GFP. Huh7 cells were fixed, stained with DAPI, and analyzed via high-content scanning. The left panel shows the images from high-content screening of GFP in the synergistic combination group, while the right panel displays DAPI staining.

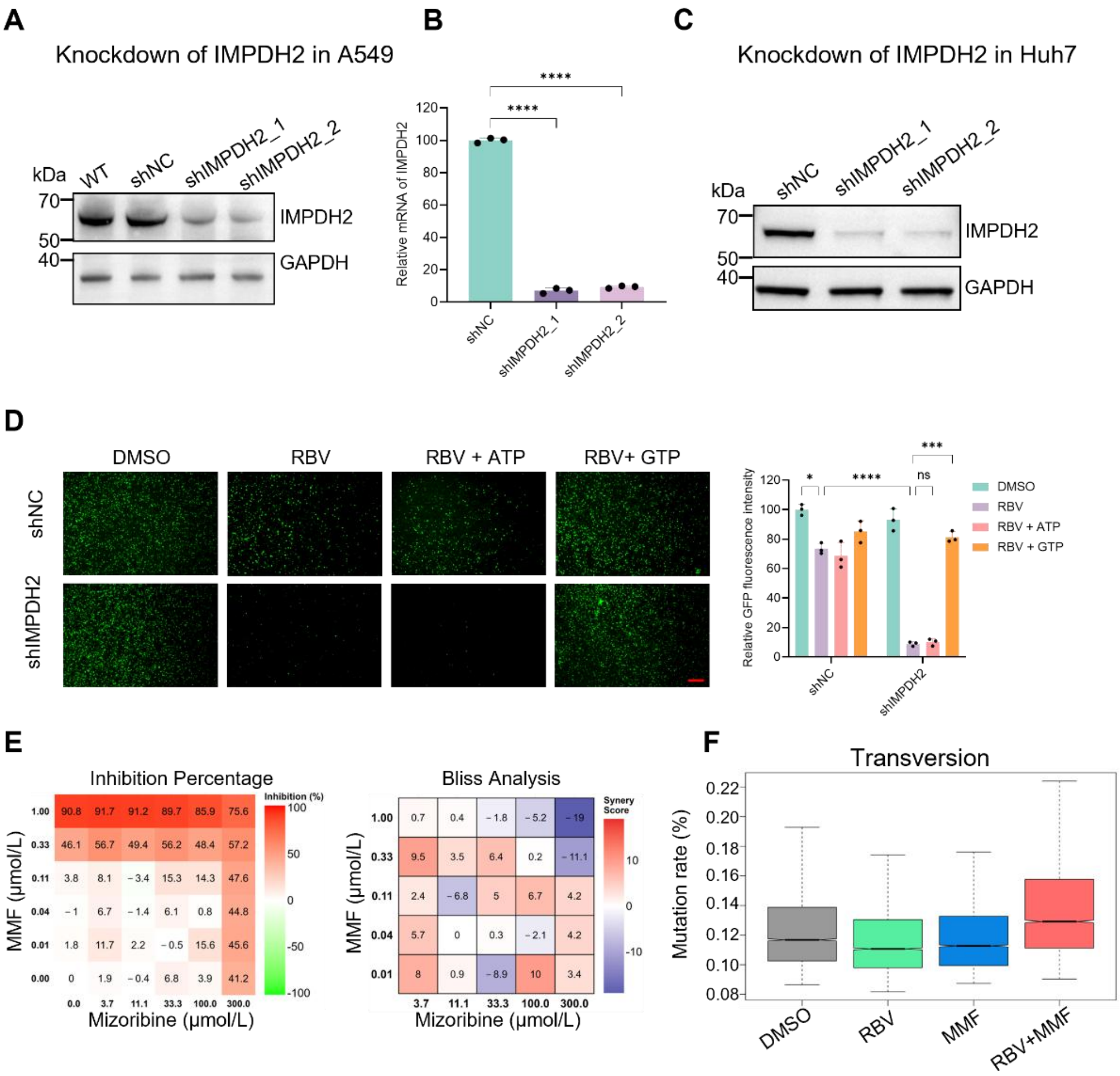

Figure S3. MMF exhibited synergistic effect with ribavirin by targeting host IMPDH2 in Huh7. (A) IMPDH2 knockdown validation in by western blot. The control group of A549 cells was either not transduced or transduced with scramble shRNA (WT and shNC). The knockdown groups were transduced with two distinct shRNAs targeting IMPDH2 (shIMPDH2_1 and shIMPDH2_2). Western blot analysis was carried out to detect the expression of IMPDH2 in A549 cells. (B) IMPDH2 knockdown validation by qPCR. The control group was transduced with shNC. The knockdown group were transduced with two distinct shRNA (shIMPDH2_1 and shIMPDH2_2). Quantitative real-time PCR was used to assess the mRNA level of *IMPDH2* in A549 cells post shRNA-mediated knockdown by lentivirus. *GAPDH* were utilized as an internal control. Data are presented as mean ± SD (n = 3 independent biological replicates) (*****P*<0.0001, one way ANOVA with Tukey's multiple comparison test). (C) IMPDH2 knockdown validation by western blot in Huh7 cells. The control group was transduced with shNC. The knockdown groups were transduced with two distinct shRNA (shIMPDH2_1 and shIMPDH2_2). Western blot analysis was performed to detect IMPDH2 expression in Huh7 cells. (D) Exogenous supplementation of GTP reversed the synergistic antiviral effect of RBV with IMPDH2 knockdown. shNC or shIMPDH2 transduced Huh7 cells were treated with or without RBV at the concentration of 20 μmol/L for 24 h, followed by treatment with 100 μmol/L ATP or

GTP. Following infection with PR8-NS1-GFP at an MOI of 0.5, GFP fluorescence was quantified at 24 h post-infection. The bar chart shows the relative GFP fluorescence intensity in each group. Scale bar, 100 μm (red). Data are presented as mean ± SD (n = 3 independent biological replicates) (***$P < 0.001$, one-way ANOVA with Tukey's multiple comparison test). (E) The synergistic effect of mizoribine with MMF against PR8-NS1-GFP in Huh7 cells. The inhibition percentage and synergy score matrix for mizoribine and MMF against PR8-NS1-GFP were assessed. Huh7 cells were treated with the indicated drugs and followed by PR8-NS1-GFP infection, cells were fixed with paraformaldehyde and stained with DAPI after 24 h post-infection. Then the cells were analyzed by high-content screen system. (F) Transversion mutation rate in PR8 vRNA. Box plots showing the transversion mutation rate in the RNA viral genome across four treatment groups: DMSO, RBV, MMF, and RBV+MMF. The y-axis represents the transversion mutation rate per nucleotide. Each box plot displays the median, interquartile range, and outliers.

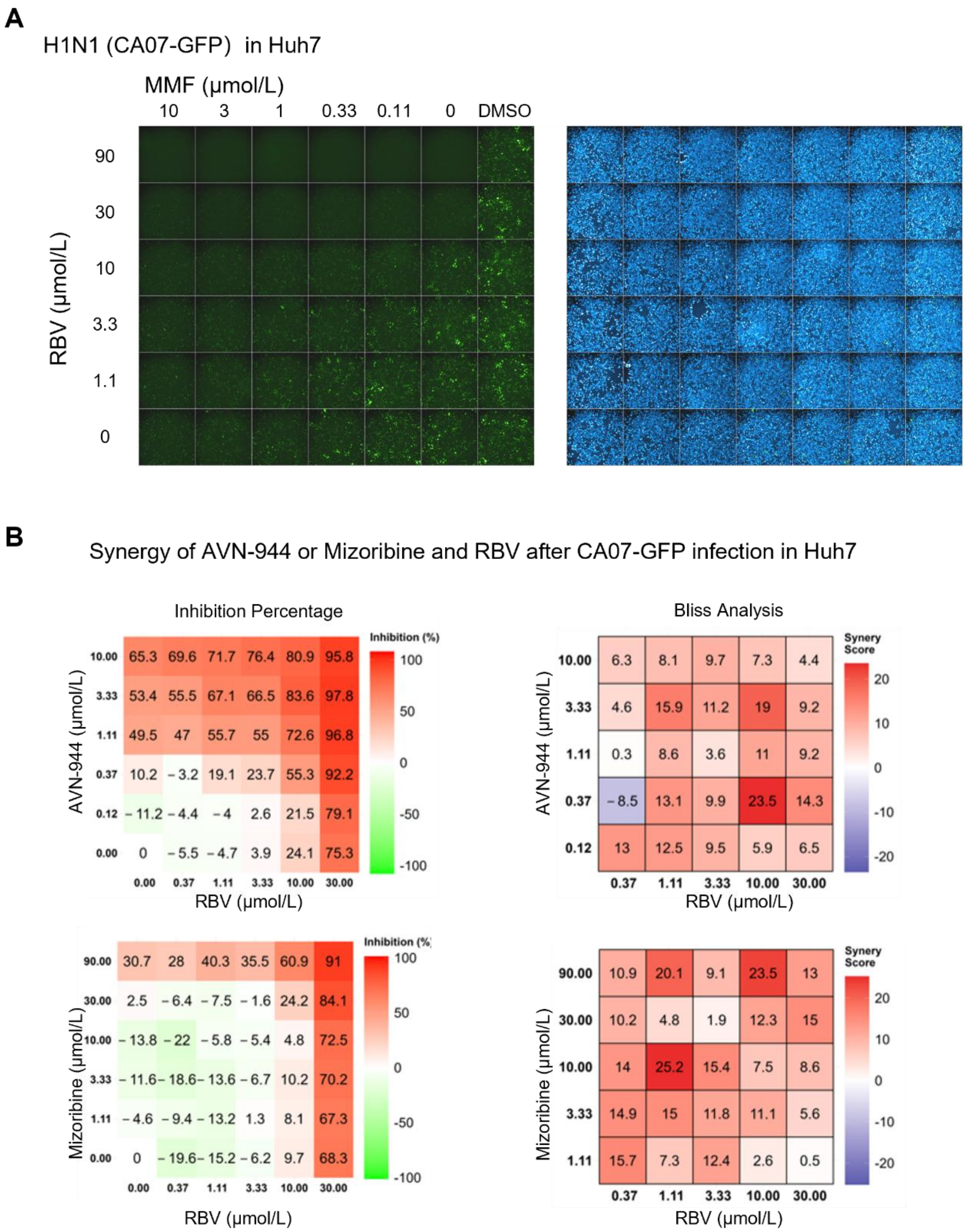

Figure S4. MMF showed synergistic effects against other H1N1 strains. (A) Synergistic effect of MMF in combination with RBV against H1N1 CA07-GFP in Huh7 cell line. Huh7 cells were treated with RBV in combination with MMF under indicated concentrations. The inhibition percentages and synergy scores were determined by quantifying the GFP fluorescence after 36 h infection with CA07-GFP. Huh7 cells were fixed, stained with DAPI, and analyzed via high-content scanning at 36 h post-infection. The left panel shows the images from high-content screening of GFP in the synergistic combination group, while the right panel displays DAPI staining. (B) Synergistic effect of AVN-944 or mizoribine in combination with RBV against H1N1 CA07-GFP in Huh7 cells. Huh7 cells were treated with RBV in combination with MMF under indicated

concentrations. The inhibition percentages and synergy scores were determined by quantifying the GFP fluorescence after 36 h infection with CA07-GFP.

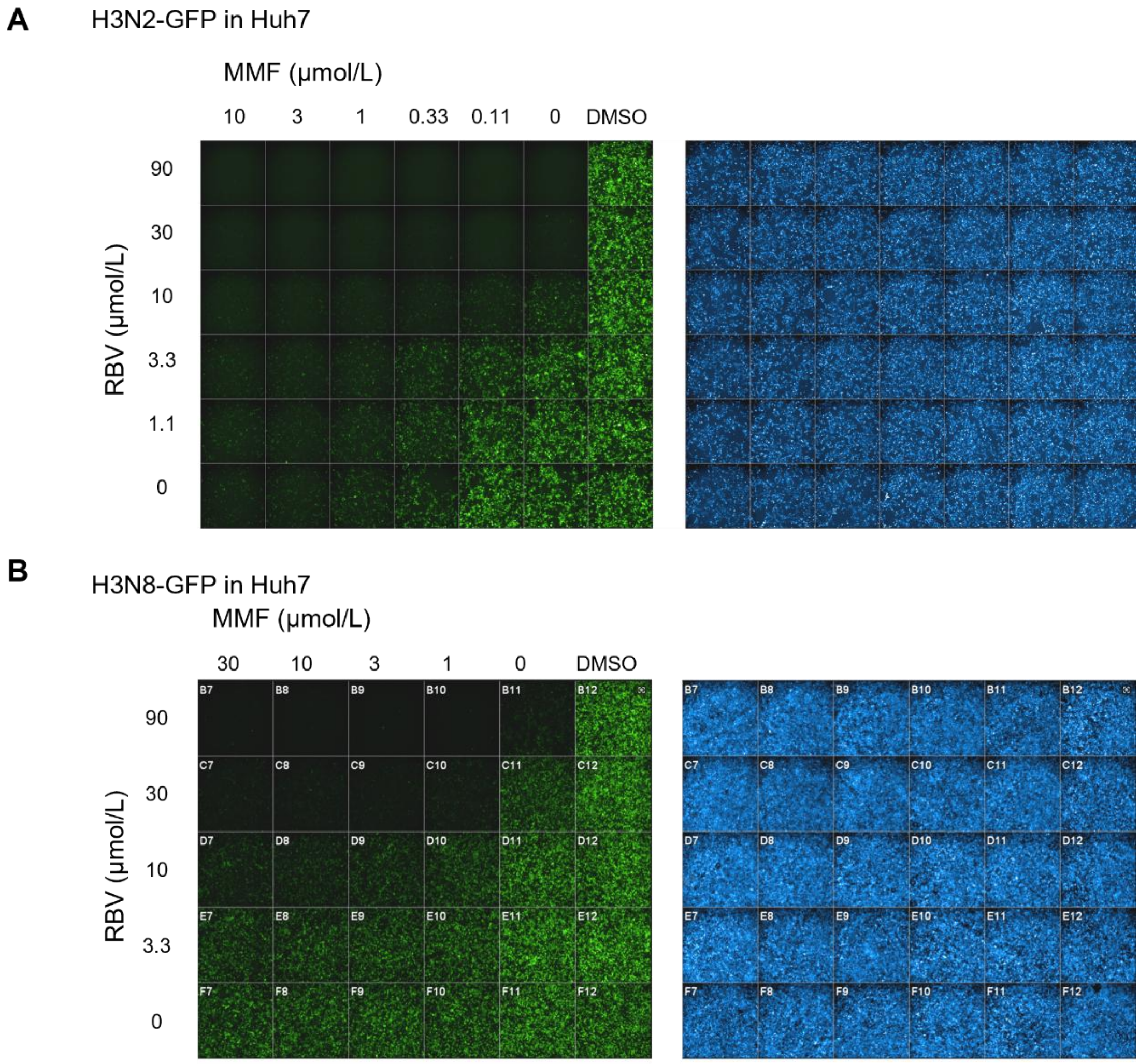

Figure S5. MMF showed synergistic effects with ribavirin against other influenza subtypes. (A) Synergistic effect of MMF in combination with RBV against H3N2-GFP in Huh7 cell line. Huh7 cells were treated with RBV in combination with MMF under indicated concentrations. The inhibition percentages and synergy scores were determined by quantifying the GFP fluorescence after 36 h infection with H3N2-GFP. Huh7 cells were fixed, stained with DAPI, and analyzed via high-content scanning. The left panel shows the images from high-content screening of GFP in the synergistic combination group, while the right panel displays DAPI staining. (B) Synergistic effect of MMF in combination with RBV against H3N8-GFP in Huh7 cell line. Huh7 cells were treated with RBV in combination with MMF under indicated concentrations. The inhibition percentages and synergy scores were determined by quantifying the GFP fluorescence after 36 h infection with H3N8 -GFP. Huh7 cells were fixed, stained with DAPI, and analyzed via high-content scanning. The left panel shows the images from high-content screening of GFP in the synergistic combination group, while the right panel displays DAPI staining.

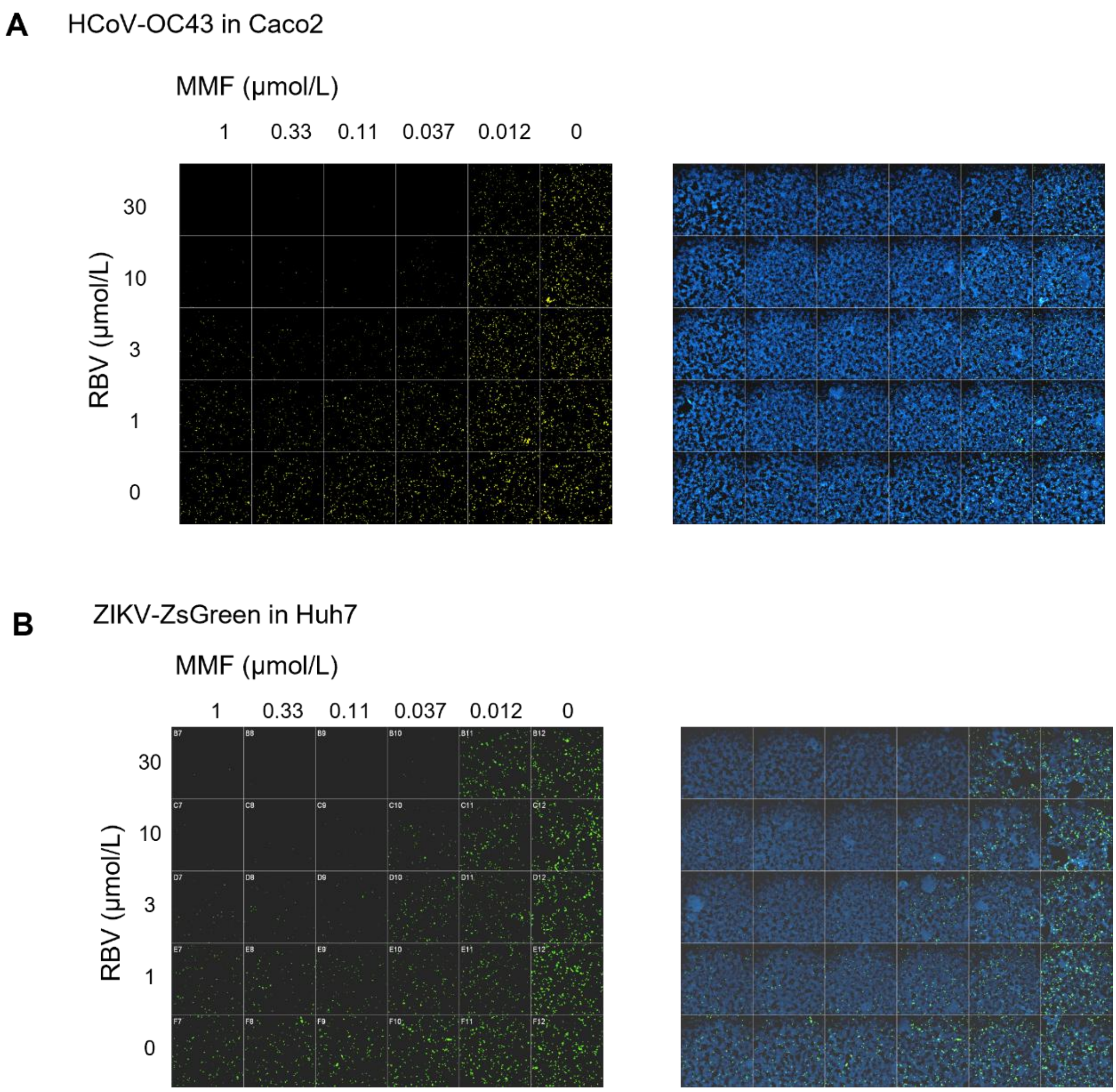

Figure S6. MMF showed synergistic effects with ribavirin against other RNA viruses. (A) Synergistic effect of MMF in combination with RBV against HCoV-OC43 in Caco2 cells. Caco2 cells were treated with RBV in combination with MMF under indicated concentrations. The inhibition percentages and synergy scores were determined by quantifying the GFP fluorescence after 72 h infection with HCoV-OC43. Caco2 cells were fixed, stained with HCoV-OC43 NP antibody and DAPI. Then the plates were analyzed via high-content scanning. The left panel shows the images from high-content screening of GFP in the synergistic combination group, while the right panel displays DAPI staining. (B) Synergistic effect of MMF in combination with RBV against Zika-ZsGreen in Huh7 cell line. Huh7 cells were treated with RBV in combination with MMF under indicated concentrations. The inhibition percentages and synergy scores were determined by quantifying the ZsGreen fluorescence after 36 h infection with Zika-ZsGreen. Huh7 cells were fixed, stained with DAPI. Then the plates were analyzed via high-content scanning. The left panel shows thumbnail images from high-content screening of GFP in the synergistic combination group, while the right panel displays DAPI staining.

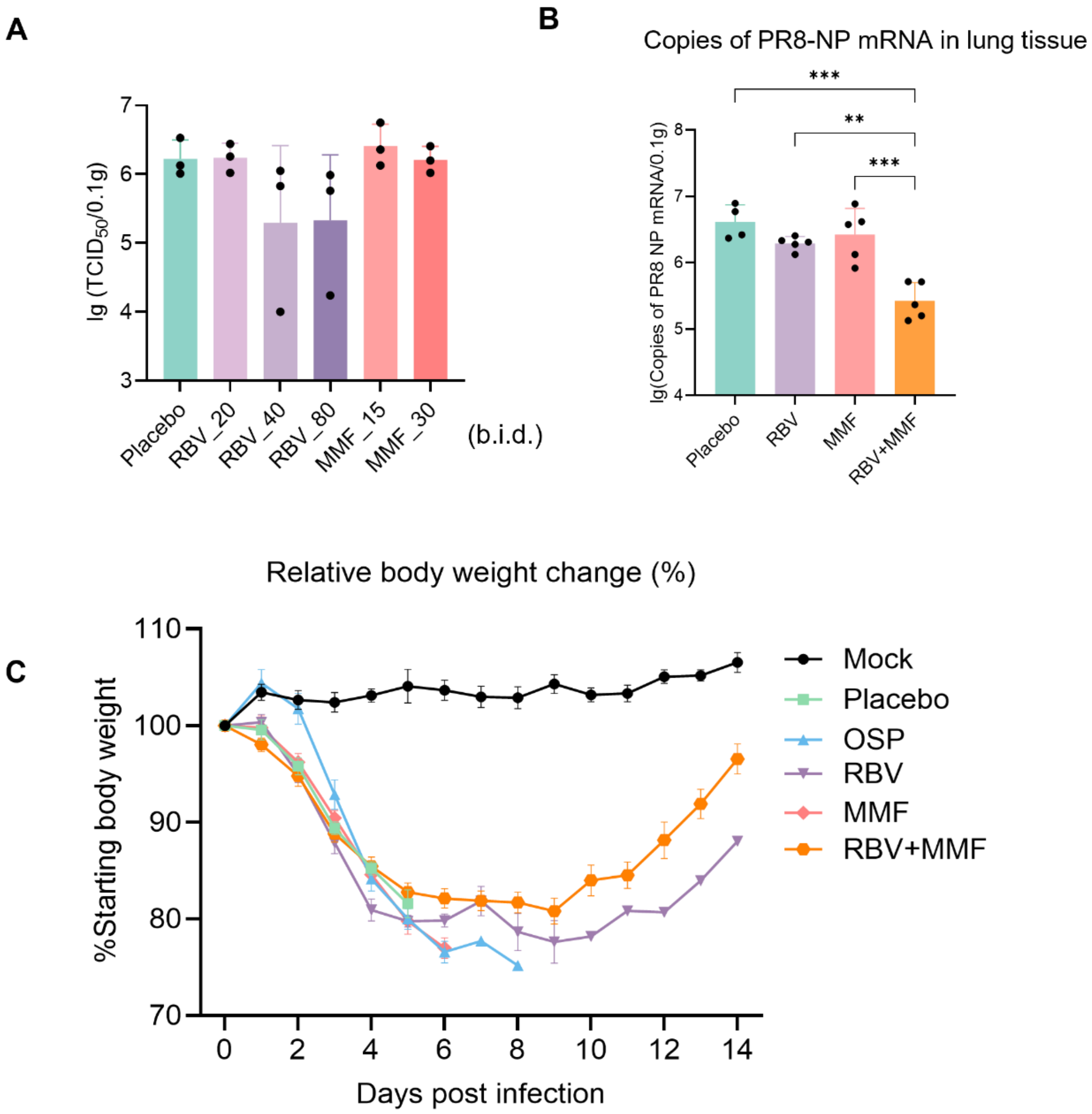

Figure S7. Synergistic antiviral effects of ribavirin and MMF in vivo. (A) Preliminary dose-ranging experiments of RBV or MMF. The compound was administered via oral gavage. RBV was tested at 20, 40, and 80 mg/kg/dose (b.i.d.), MMF was tested at 15 and 30 mg/kg/dose (b.i.d.). Then the mice (n = 3) was chanllenged with PR8. Mouse lung tissues were collected and homogenized for viral titer quantification at 4 days post-infection. (B) Determination of viral genome copies in lung tissue by qPCR. Mouse lung tissues were collected and homogenized for viral genome quantification at 4 days post-infection. Mean ± SD is shown. $^{**}P < 0.01$, ns $P > 0.05$, using one-way ANOVA with Tukey's multiple comparison test. (C) Fourteen-day *in vivo* evaluation of the antiviral efficacy of combined RBV and MMF treatment in a mouse model. Female BALB/c mice (6-8 weeks) were administrated with PBS (Placebo, n=4), oseltamivir phosphate (10 mg/kg, b.i.d., n = 5), RBV (40 mg/kg, b.i.d., n=5), MMF (15 mg/kg, b.i.d., n=5), or the combination (RBV 40 mg/kg, b.i.d. + MMF 15 mg/kg, b.i.d., n = 5) via oral gavage 2 h prior to intranasal challenge with 1000 PFU PR8 (H1N1). Mice were monitored daily for body weight changes over a 14-day period post-infection.

Table S1. Primers for RNA Quantification of mice tissue, human cell lines and viral genome.

**Species**	**Gene**	**Primer Sequence**
Mouse	*ISG15*	F: CATCCTGGTGAGGAACGAAAGG<br>R: CTCAGCCAGAACTGGTCTTCGT
	Mx1	F: TGGACATTGCTACCACAGAGGC<br>R: TTGCCTTCAGCACCTCTGTCCA
	CXCL10	F: ATCATCCCTGCGAGCCTATCCT<br>R: GACCTTTTTTGGCTAAACGCTTTC
	1L-1β	F: ACTGTTTCTAATGCCTTCCC<br>R: ATGGTTTCTTGTGACCCTGA
	TNF	F: GAACTGGCAGAAGAGGCACT<br>R: AGGGTCTGGGCCATAGAACT
	CCL2	F: CTTCTGGGCCTGCTGTTCA<br>R: CCAGCCTACTCATTGGGATCA
Human	*IMPDH2*	F: AGTGGCTCCATCTGCATTACGC<br>R: GGATTCCTCCATCAGCAATGACC
IAV	*PR8-NP*	F: TGTGTATGGACCTGCCGTAGC<br>R: CCATCCACACCAGTTGACTCTTG

Table S2. shRNA sequences targeting IMPDH2 gene and control.

Gene	F/R	Oligo Sequence
shIMPDH2_1	Forward oligo	CCGGCACCTACAATGACTTTCTCATCTCGAGATGAGAAAGTCATTGTAGGTGTTTTTG
	Reverse oligo	AATTCAAAAACACCTACAATGACTTTCTCATCTCGAGATGAGAAAGTCATTGTAGGTG
shIMPDH2_2	Forward oligo	CCGGGACTGTTTCTTGGAAGAGATACTCGAGTATCTCTTCCAAGAAACAGTCTTTTTG
	Reverse oligo	AATTCAAAAAGACTGTTTCTTGGAAGAGATACTCGAGTATCTCTTCCAAGAAACAGTC
shNC	Forward oligo	CCGGCAACAAGATGAAGAGCACCAACTCGAGTTGGTGCTCTTCATCTTGTTGTTTTTG
	Reverse oligo	AATTCAAAAACAACAAGATGAAGAGCACCAACTCGAGTTGGTGCTCTTCATCTTGTTG
